# The Earliest Chinese Proto-Porcelain Excavated from Kiln Sites: An Elemental Analysis

**DOI:** 10.1371/journal.pone.0139970

**Published:** 2015-11-04

**Authors:** Yu Li, Bin Zhang, Huansheng Cheng, Jianming Zheng

**Affiliations:** 1 Applied Ion Beam Physics Laboratory (Key Laboratory of the Ministry of Education), Institute of Modern Physics, Fudan University, Shanghai, People's Republic of China; 2 Zhejiang Provincial Institute of Cultural Relics and Archaeology, Hangzhou, Zhejiang, People's Republic of China; New York State Museum, UNITED STATES

## Abstract

In June 2012, the Piaoshan kiln site was excavated in Huzhou, Zhejiang Province, which hitherto proved to be the earliest known Chinese proto-porcelain kiln. Judging from the decorative patterns of unearthed impressed stoneware and proto-porcelain sherds, the site was determined to date to the late Xia (c. 2070–c. 1600 BC), the first dynasty of China. Here, we report on proton-induced X-ray emission analyses of 118 proto-porcelain and 35 impressed stoneware sherds from Piaoshan and five subsequent kiln sites in the vicinity. Using principal components analysis on the major chemical compositions, we reveal the relationships between impressed stoneware and proto-porcelain samples from the six kiln sites. The sherds from different sites have distinctive chemical profiles. The results indicate that the raw materials were procured locally. We find a developmental tendency for early glazes towards mature calcium-based glaze. It is most likely that woody plant ashes with increased calcia-potash ratios were applied to the formula.

## Introduction

China has a long history of making pottery and ceramics. The making of pottery in China dates back to the Upper Paleolithic era, about 20,000 BP [1]. Along with the maturity of pottery making technology, especially the development of impressed stoneware, came proto-porcelain, the primitive stage production of Chinese porcelain [2]. In 1929, the excavation of Yinxu, Anyang, a Shang capital in Henan province, revealed some unprecedented early ceramics with high hardness and low water absorption. Attempts to evaluate their status in the ceramic history were met with difficulties due to their novelty and rarity. At that time, they could only be described temporarily as “glazed pottery” [3]. During the following decades, more “glazed pottery” was unearthed from tombs and residential sites from across China, and people came to realize that the primitive glazed ceramics had already been produced at such an early age [4]. As a result, the idea of proto-porcelain was firstly introduced in the 1960s [5].

According to the understanding of archaeological materials, scholars previously asserted that proto-porcelain originated in the Shang dynasty (c. 1600–1046 BC) and developed during the Zhou dynasty (c. 1046–256 BC) [6][7]. However, with the discovery of the Erlitou Site in Yanshi, Henan province, the earliest physical evidence of proto-porcelain emerged from the legendary Xia (c. 2070–c. 1600 BC), the first dynasty of China [8][9]. Unfortunately, corresponding kilns from such an early time period had never been discovered.

There has been a longstanding controversy about the regional origin of proto-porcelain, regarding whether it was produced in southern China and then traded to the north, or produced in southern and northern China respectively. The related typological styles of some proto-porcelain unearthed in northern China and local pottery seem to support evidence for the latter proposition [10][11][12]. Nevertheless, the similarity of chemical compositions reflected the relationship of inheritance between proto-porcelain and later southern porcelain, which could not be found between proto-porcelain and later northern porcelain [13][14][15]. Moreover, to date no proto-porcelain kiln sites have been found in northern China. Relevant studies are, however, somewhat limited by the lack of systematic analysis of proto-porcelain excavated from tombs, residential sites and in kilns [16].

**Fig 1:**
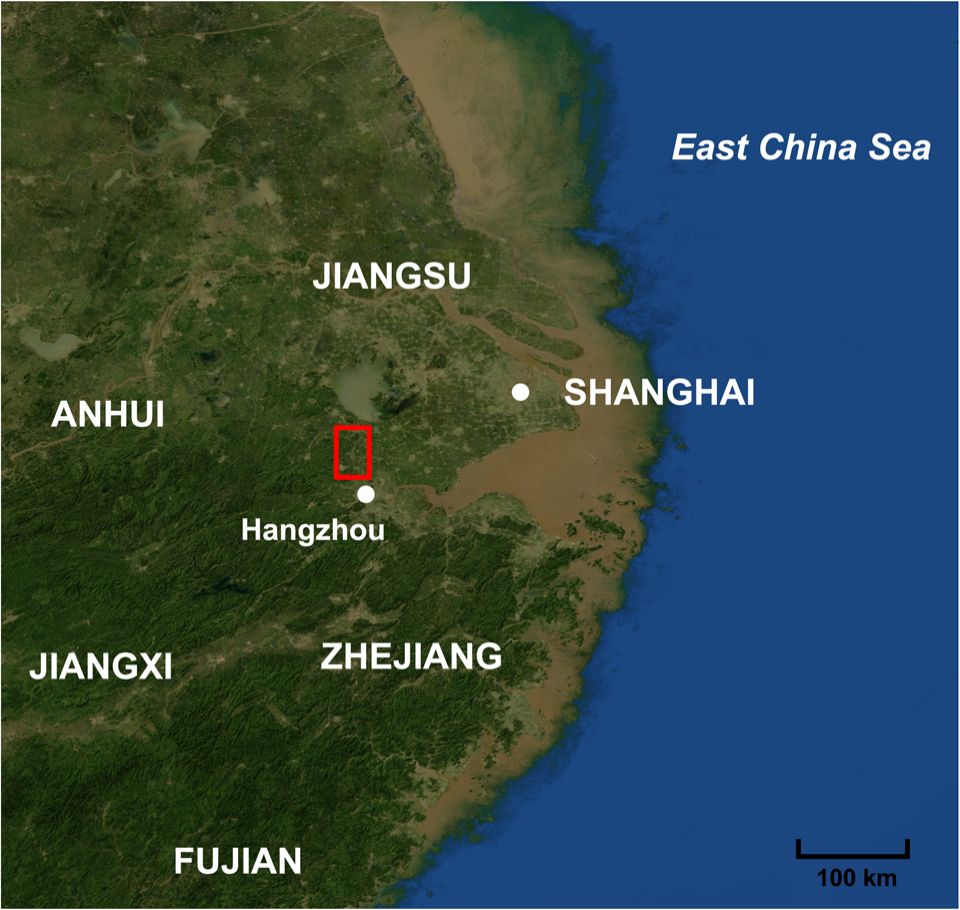
Map of the location of Huzhou and Deqing. The red square indicates the area in which the six kiln sites are located. The base map created using the Blue Marble Next Generation with Topography and Bathymetry, July (http://visibleearth.nasa.gov/view.php?id=73751) [23].

**Fig 2:**
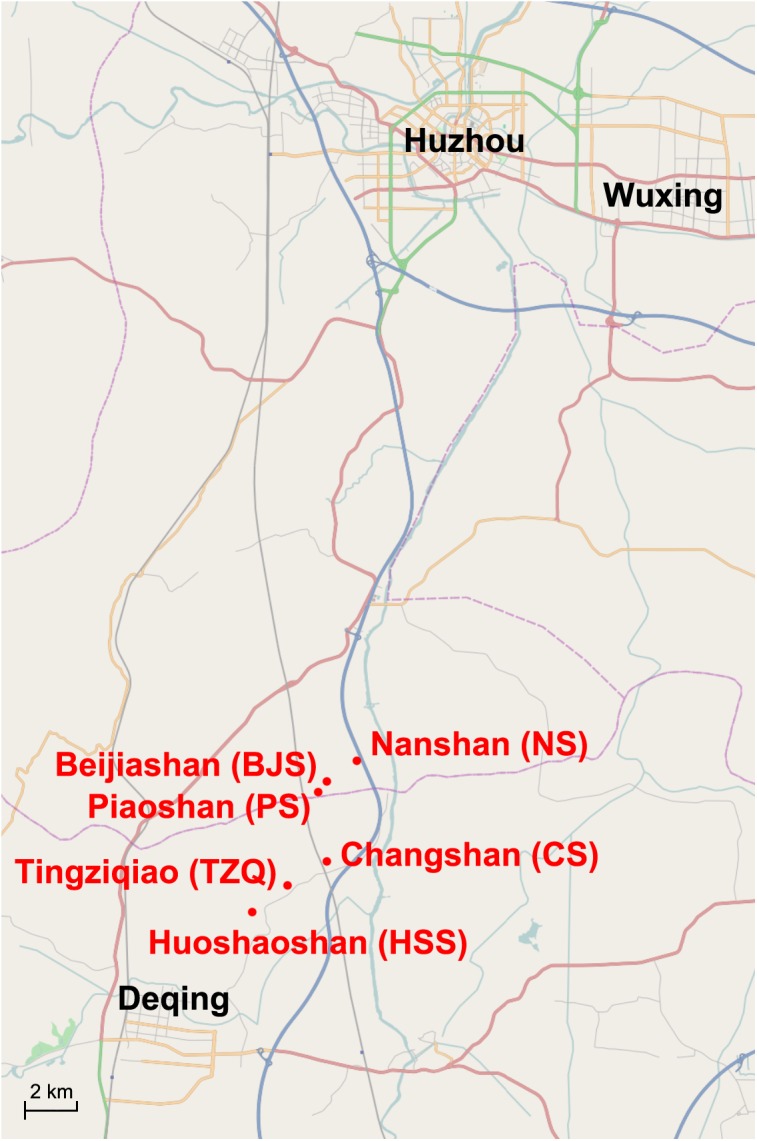
The geographical locations of six kilns in Huzhou and Deqing in Zhejiang province. The red spots indicate the six kilns mentioned in the text. The base map created using OpenStreetMap, shared under the Open Database Licence (http://www.opendatacommons.org/licenses/odbl).

In recent years, a group of kiln sites of the pre-Qin period were discovered in Zhejiang province (Figs 1 and 2) [17][18][19]. In June 2012, the Piaoshan kiln site was excavated in Huzhou, Zhejiang province, which proved to be the earliest proto-porcelain kiln ever discovered [20][21]. It is a kiln that produced impressed stoneware and proto-porcelain during the same period. Judging from current archaeological materials, the zigzag patterns on the impressed stoneware are considered an imitation of bamboo-woven objects. These patterns were popular between the late Neolithic era and Xia–Shang dynasty [22]. The impressed stoneware unearthed in Piaoshan kiln site was therefore inferred to be from the late Xia dynasty.

For more than 35 years, an accelerator laboratory has been operated at Fudan University for the study of archaeometry. Since the nationally renowned ion beam analysis of Yue Goujian Sword [24], we have accumulated a large amount of compositional data about ancient Chinese jade, glass, pottery and ceramics [25][26][27][28]. In this paper, we investigate the origin and early development of proto-porcelain by analysing samples from three of the earliest Chinese proto-porcelain kiln sites in Huzhou, Zhejiang province, together with samples from three later kiln sites in nearby Deqing, Zhejiang province. In particular, the chemical composition of proto-porcelain samples from the earliest Piaoshan kiln site of the Xia dynasty is studied for the first time.

## Materials and Methods

No permits were required for the described study, which complied with all relevant regulations. As shown detailedly in Table 1, the samples selected for this study consist of 153 sherd specimens (proto-porcelain: n = 118; impressed stoneware: n = 35) excavated from six kiln sites (S1–S3 Figs): Piaoshan (PS), Beijiashan (BJS), Nanshan (NS), Huoshaoshan (HSS), Changshan (CS), and Tingziqiao (TZQ). All sherd specimens are publicly deposited in Zhejiang Provincial Institute of Cultural Relics and Archaeology, Hangzhou, Zhejiang province. The Piaoshan, Beijiashan, and Nanshan kiln sites are located in the southern suburb of Huzhou City, Zhejiang province (Fig 2). Comparison kiln sites at Huoshaoshan, Tingziqiao and Changshan are located in Deqing County, about 30 km away from the south of Huzhou City. All the samples were catalogued during the excavation by Zhejiang Provincial Institute of Cultural Relics and Archaeology.

**Table 1:** Basic information of the samples from six kiln sites at Huzhou and Deqing, Zhejiang province.

Name of the sites	Time periods	Samples	*n*1	*n*2
Piaoshan (PS)	Late Xia	17 sherds	15	2
Beijiashan (BJS)	Xia–Shang	16 sherds	8	8
Nanshan (NS)	Early Shang	20 sherds	9	11
Early Huoshaoshan (HSS1)	Late Western Zhou–Early Spring and Autumn period (L.WZ–E.S&A)	20 sherds	0	20
Middle Huoshaoshan (HSS2)	Middle Spring and Autumn period (M.S&A)	20 sherds	0	20
Late Huoshaoshan (HSS3)	Late Spring and Autumn period (L.S&A)	20 sherds	0	20
Changshan (CS)	Early Warring States period (E.WS)	20 sherds	1	19
Tingziqiao (TZQ)	Early Warring States period (E.WS)	20 sherds	2	18

*n*1: the number of sherds of impressed stoneware from each site

*n*2: the number of proto-porcelain sherds from each site.

The proton-induced X-ray emission (PIXE) experiments were performed on the NEC 9SDH-2 3 MV pelletron tandem accelerator at Fudan University. An external beam line was applied to determine chemical compositions of the proto-porcelain samples. The proton beam was collimated with a spot diameter of 1mm and then emerged through a 7.5 μm Kapton film into the air. In order to keep the dead time lower than 3%, the beam current was maintained at 0.05 nA. After travelling about 10 mm, the beam hit the sample. Due to the energy loss in the Kapton film and the air, the initial 3 MeV protons are attenuated to 2.8 MeV. The PIXE spectra were collected using a SGX Sensortech Si (Li) detector (with an energy resolution of 150 eV FWHM at 5.9 keV) placed perpendicularly to the beam direction. The X-rays emitted from the sample travelled through a helium-rich atmosphere before reaching the detector. Details of the experimental procedure have been reported elsewhere [29][30]. One well-established geochemical standard reference sample GSD-6 from National Experimental Analysis Center of Chinese Geological Ministry [31][32][33], was used to determine experimental parameters. Data treatment was made with the GUPIX-96 code [34].

Non-uniform thickness of the glaze posed a great challenge to the measurements of the elemental composition of the glaze. The thinnest glaze is about 100 μm, while the projected range for a 2.8 MeV proton beam in typical porcelain glazes is about 130 μm [35]. These locations were not suitable for analysing the elemental composition of the glaze since it is difficult to know whether the X-rays detected were due to the glaze or the body further down. Theoretical values of the information depth for PIXE measurements in a ceramic sherd were estimated for a signal fraction of 97% for various X-ray energy levels. The energy levels for K Kα line, Ca Kα line and Fe Kα line are 3.3 keV, 3.7 keV, and 6.4 keV respectively and X-rays at these energy levels can penetrate a depth of about 21 μm, 23 μm and 31 μm respectively. Therefore, the quantification of these main components of the glaze is possible as long as the ideal measuring points, where the glaze is thick enough (>130 μm), were selected for PIXE measurements. These measuring points must be bigger than the proton beam spot in sizes as well.

## Results

Table 2 shows the average chemical compositions of the bodies of impressed stoneware and proto-porcelain determined by PIXE (S1 Table). Owing to the fact that all these kilns produced impressed stoneware and proto-porcelain during the same period, it is reasonable to assert that, for the bodies, impressed stoneware and proto-porcelain produced from the same kiln shared the same raw materials [36]. For that reason, we take impressed stoneware into account when analysing the chemical compositions of the bodies. Table 3 shows the average chemical compositions of the glazes on the proto-porcelain for each kiln site (S2 Table). All kiln sites are presented in chronological order. The elements are converted to corresponding oxides and then normalized to 100%.

**Table 2:** PIXE results of the average chemical compositions (wt%) of the bodies of impressed stoneware and proto-porcelain sherds from 6 kiln sites.

Sample	Date	*n*1	*n*2	*n*3	Na_2_O	MgO	Al_2_O_3_	SiO_2_	P_2_O_5_	K_2_O	CaO	TiO_2_	MnO	Fe_2_O_3_	Total
PS	L.Xia	2	15	17	0.25	0.68	18.12	73.04	0.14	1.98	0.30	1.09	0.03	4.29	99.92
BJS	Xia–Shang	8	8	16	0.60	0.61	16.26	75.55	0.36	1.89	0.38	0.96	0.03	3.32	99.95
NS	E.Shang	11	9	20	1.03	0.54	18.66	73.04	0.26	2.52	0.41	0.92	0.03	2.53	99.93
HSS1	L.WZ–E.S&A	20	0	20	0.41	0.57	18.06	75.13	0.27	2.09	0.27	0.94	0.01	2.17	99.93
HSS2	M.S&A	20	0	20	0.63	0.49	16.63	76.22	0.11	2.39	0.36	0.88	0.02	2.21	99.95
HSS3	L.S&A	20	0	20	0.82	0.43	16.27	75.97	0.15	2.82	0.39	0.97	0.03	2.09	99.94
CS	E.WS	19	1	20	0.64	0.54	15.29	77.94	0.15	1.79	0.38	1.04	0.02	2.18	99.96
TZQ	E.WS	18	2	20	0.42	0.61	15.59	77.21	0.19	1.81	0.36	1.14	0.02	2.59	99.95

*n*1: the number of proto-porcelain sherds from each site

*n*2: the number of sherds of impressed stoneware from each site

*n*3: the number of the sherds from each site. The low analytical totals are due to the porosity of the ceramic.

**Table 3:** PIXE results of the average chemical compositions (wt%) of the glazes on proto-porcelain sherds from 6 kiln sites.

Sample	Date	*n*1	*n*2	*n*3	Na_2_O	MgO	Al_2_O_3_	SiO_2_	P_2_O_5_	K_2_O	CaO	TiO_2_	MnO	Fe_2_O_3_	Total
PS	L.Xia	2	21	17	0.51	1.40	15.29	66.49	0.37	3.37	6.00	1.02	0.14	5.28	99.85
BJS	Xia–Shang	9	19	16	0.83	1.44	14.74	66.58	0.60	2.68	7.50	0.93	0.16	4.50	99.93
NS	E.Shang	11	25	20	1.16	1.33	15.10	62.56	0.69	3.11	10.73	1.06	0.20	3.71	99.66
HSS1	L.WZ–E.S&A	23	23	20	0.57	1.95	15.00	58.70	0.96	2.83	14.80	0.78	0.12	4.17	99.88
HSS2	M.S&A	20	20	20	0.76	1.98	13.76	63.44	1.10	2.68	12.60	0.83	0.26	2.50	99.92
HSS3	L.S&A	20	20	20	0.97	1.84	13.91	62.85	0.96	2.76	12.92	0.91	0.27	2.54	99.92
CS	E.WS	19	19	20	0.69	2.60	11.77	60.09	1.36	1.79	17.70	0.87	0.42	2.58	99.86
TZQ	E.WS	18	20	20	0.49	2.18	12.01	59.89	1.08	2.08	17.09	1.00	0.53	3.51	99.88

*n*1: the number of areas considered to be glazed of all the sherds from each site

*n*2: the number of areas analyzed of all the sherds from each site

*n*3: the number of the sherds from each site.

### The bodies

The content of titanium dioxide ranges from 0.88 wt% to 1.14 wt%, while the dominant silicon dioxide and aluminium(III) oxide account for 73.04–77.94 wt% and 15.29–18.66 wt%, respectively. For sodium, magnesium, phosphorus, calcium and manganese, the concentration of their oxides are less than or approximately 1 wt%. The results show that these samples belong to typical southern ceramic body system in which porcelain stone is used as the raw material [37][38]. Low-aluminum (<20 wt%) and high-titanium (about 1 wt%) features were still obvious for porcelain products made in southern China even after two thousand years, when southern porcelain developed into a much more mature stage during the Tang and Song dynasties [39][40][41]. Note that the earliest Piaoshan samples revealed considerably high level (4.29 wt%) of iron(III) oxide, suggesting the raw material selection was in its very early stage at that time. Regarding samples from three of the earliest proto-porcelain kilns, there is a significant decline in iron(III) oxide chronologically from 4.29 wt% to 2.53 wt%, while the other late samples remain at a 2 wt% level. This indicates that porcelain stone used as the raw material was carefully selected and filtered to remove iron-rich particulate impurities, as technology developed. The quality improvement is visually expressed by higher whiteness of the body.

### The glazes

The glazes are also dominated by silicon dioxide (59.89–66.58 wt%) and aluminium(III) oxide (11.77–15.29 wt%), but their proportions are lower than those in the bodies. Due to the rareness of early proto-porcelain, only two Piaoshan proto-porcelain samples were collected for PIXE analysis. The obvious high calcium oxide level (4.92 wt% and 7.08 wt%) marked the earliest attempt of applying artificial calcium-based glaze. In Table 3, the historical development of proto-porcelain glazes is highlighted by an increasing utilisation of calcium oxide (from 6.00 wt% to 17.70 wt%) and a decreasing trend for potassium oxide (from 3.37 wt% to 1.79 wt%), correspondingly and simultaneously. In concrete terms, the calcium oxide contents were initially 6–10 wt% during the Xia and Shang period, then maintained a relatively stable level of 12–14 wt% in the Spring and Autumn period, and finally increased to 17–18 wt% in the Warring States period. This change indicates that the amount of calcium-rich material, which was purposely added to the formula composition of the glazes, increased over time.

In particular, among all BJS samples, the calcium oxide contents of two samples are much higher than the average 4–6 wt% level, reaching 17.36 wt% and 14.07 wt%, which are quite close to that of later period, giving direct evidence that the earliest transition started in somewhere between the Xia and Shang dynasties. The contents of phosphorus(V) oxide (0.37–1.36 wt%) and magnesium oxide (1.31–2.60 wt%) were also increasing over time and were higher than that of the body, suggesting phosphorus–rich and magnesium–rich plant ashes were added to the formula composition of the glazes.

### Principal components analysis

The chemical composition reveals little information directly, therefore it is beneficial to conduct multivariate statistical analysis in the areas of archaeometry. Principal components analysis (PCA) is one of the multivariate statistical methods that performs well in solving problems of material identification and provenance determination [42][43][44].

**Fig 3:**
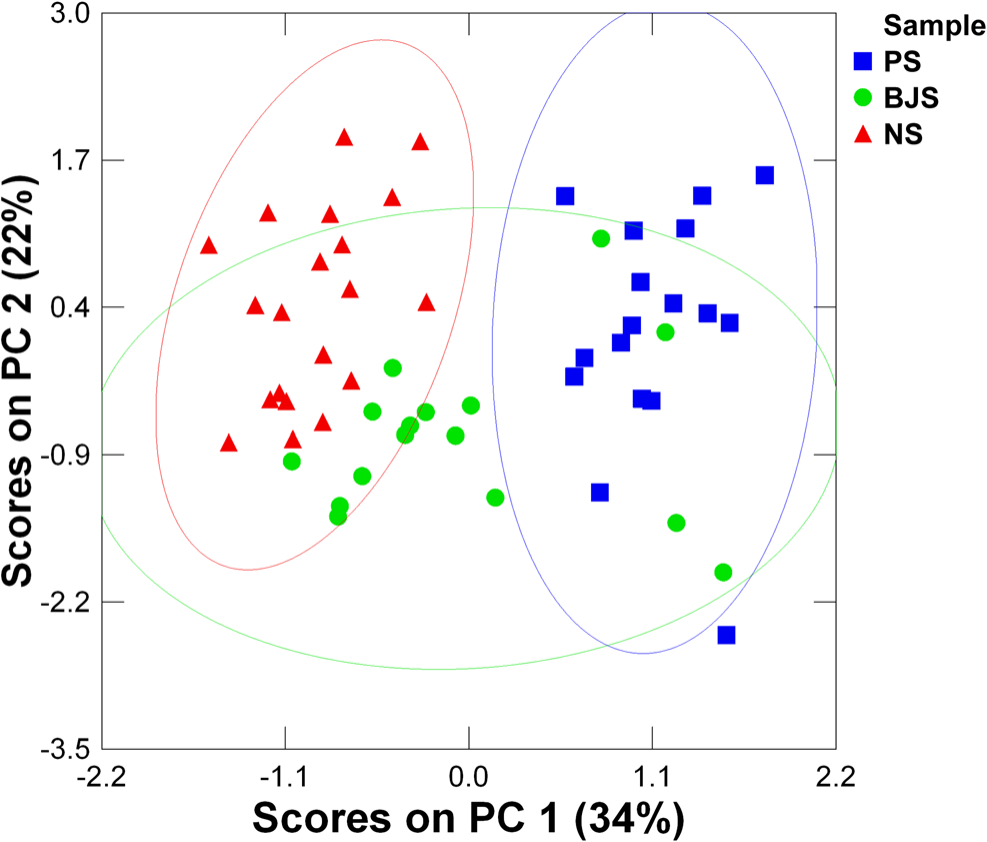
The diagram of principal components analysis on bodies of impressed stoneware and proto-porcelain from three Huzhou kiln sites. The separated clusters indicate that the raw materials of bodies of impressed stoneware and proto-porcelain from Piaoshan, Beijiashan and Nanshan were from different locations and procured locally. The solid line ellipses represent the 95% confidence limit for each cluster respectively.
$$\mathrm{PC}1=-0.924{\mathrm{Na}}_{2}O+0.543\mathrm{MgO}+0.014{\mathrm{Al}}_{2}O_{3}-0.152\mathrm{Si}O_{2}-0.243P_{2}O_{5}-0.600K_{2}O-0.464\mathrm{CaO}+0.801\mathrm{Ti}O_{2}+0.403\mathrm{MnO}+0.897{\mathrm{Fe}}_{2}O_{3}$$
$$\mathrm{PC}2=0.106{\mathrm{Na}}_{2}O+0.405\mathrm{MgO}+0.901{\mathrm{Al}}_{2}O_{3}-0.960\mathrm{Si}O_{2}-0.187P_{2}O_{5}+0.281K_{2}O+0.334\mathrm{CaO}-0.014\mathrm{Ti}O_{2}-0.205\mathrm{MnO}+0.102{\mathrm{Fe}}_{2}O_{3}$$

Fig 3 shows the results for a PCA performed on chemical compositions of impressed stoneware and proto-porcelain bodies from three kiln sites in Huzhou. Three clusters, representing Piaoshan, Beijiashan and Nanshan samples respectively, are nicely separated. It is evident that the raw materials used by these kilns came from different locations. Considering that the three kiln sites in Huzhou were closely distributed in a small geographical area, it is reasonable to infer that they were collected locally. The subtle differences between these raw materials may arise from diverse geological conditions, varying technical requirements and/or artificial selections in different kilns. Compared to late Nanshan samples, the earliest Piaoshan proto-porcelain reveal an entirely different formula composition of the bodies. For Beijiashan proto-porcelain chronologically in between, the majority of them are more closely related to late Nanshan samples. Only four samples fall into the cluster of Piaoshan proto-porcelain, attributed to high contents of iron(III) oxide (>4 wt%) because of immature raw material selection in early stage. It provides contextual information about the early development of proto-porcelain bodies.

**Fig 4:**
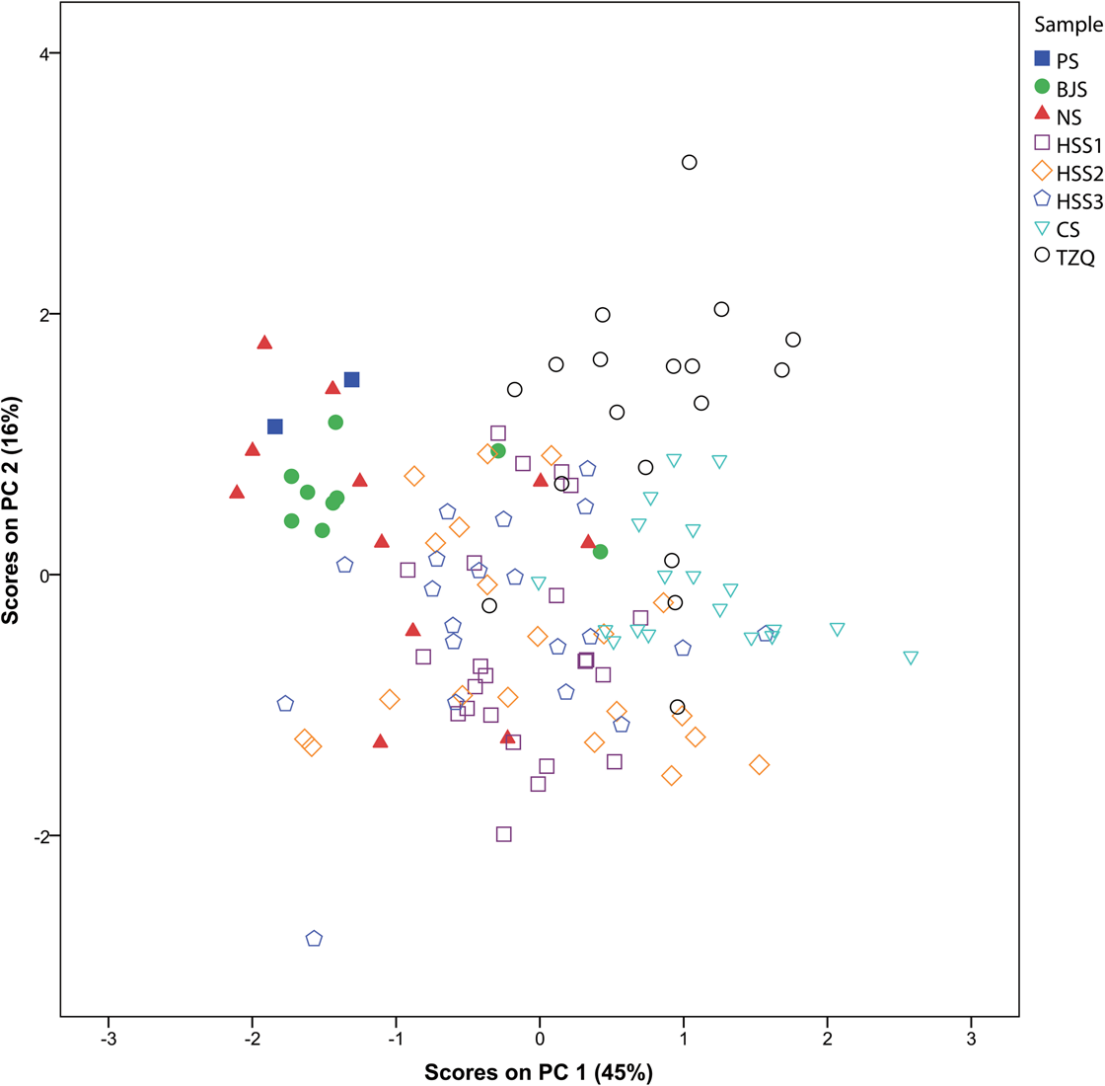
The diagram of principal components analysis on proto-porcelain glazes from six kiln sites. All the samples from the right-side cluster reach a level of more than 12 wt% in calcium oxide, while the small cluster representing early Huzhou samples is characterized by lower calcium oxide content (< 10 wt%). The confidence ellipses representing the 95% confidence limit for samples from each kiln respectively are shown in S4 Fig.
$$\mathrm{PC}1=-0.417{\mathrm{Na}}_{2}O+0.814\mathrm{MgO}-0.744{\mathrm{Al}}_{2}O_{3}-0.718\mathrm{Si}O_{2}+0.814P_{2}O_{5}-0.714K_{2}O+0.919\mathrm{CaO}-0.242\mathrm{Ti}O_{2}+0.648\mathrm{MnO}-0.320{\mathrm{Fe}}_{2}O_{3}$$
$$\mathrm{PC}2=-0.468{\mathrm{Na}}_{2}O-0.273\mathrm{MgO}-0.330{\mathrm{Al}}_{2}O_{3}+0.213\mathrm{Si}O_{2}-0.224P_{2}O_{5}-0.319K_{2}O-0.048\mathrm{CaO}+0.774\mathrm{Ti}O_{2}+0.410\mathrm{MnO}+0.455{\mathrm{Fe}}_{2}O_{3}$$

A PCA was conducted on the entire dataset consisting of 122 proto-porcelain glazes from the six kilns. Consistent with earlier discussion, the most significant difference lies in the content of calcium oxide. As shown in Fig 4, two clusters representing Huzhou and Deqing respectively are separated. Containing glazes of Piaoshan samples (2), most of the Beijiashan samples (7 of 9), and about half of the Nanshan samples (6 of 11), the small cluster is characterized by lower calcium oxide content. The relationship between the glazes from Huoshaoshan samples at three historical stages has been investigated in detail before this research [45]. Recently, the glaze of Tingziqiao proto-porcelain has also been studied [46][47][48]. In addition to the common calcium-rich feature, there has been a simultaneous tendency to increase the contents of iron(III) oxide and manganese(IV) oxide in late Deqing proto-porcelain samples. Based on both of these metrics, it should be noted that the content values of Changshan glaze are somewhere between that of late Huoshaoshan glaze and Tingziqiao glaze. For that reason, Changshan kiln was likely to produce proto-porcelain earlier than Tingziqiao kiln, despite the fact that they were both archaeologically considered to be of the Warring States period.

As for the glazes in Fig 4, a significant correlation is noticed between the Piaoshan samples and most of the Bejiashan samples (7 out of 9). Note that the two remaining Beijiashan samples are located in the right-side area of Fig 4, resulted from much higher calcium oxide contents as mentioned earlier. And for the same reasons, late Nanshan samples are split into two clusters with nearly equal number of items (5 and 6). All the samples from the right-side cluster reach a level of more than 12 wt% in calcium oxide. The original glaze of proto-porcelain was characterized by a formula composition with calcium oxide content less than 8 wt%. Then the historical development trended to calcium-rich glazes, which finally became mainstream in Shang dynasty.

## Discussion

### Selection of the raw materials of the bodies

**Fig 5:**
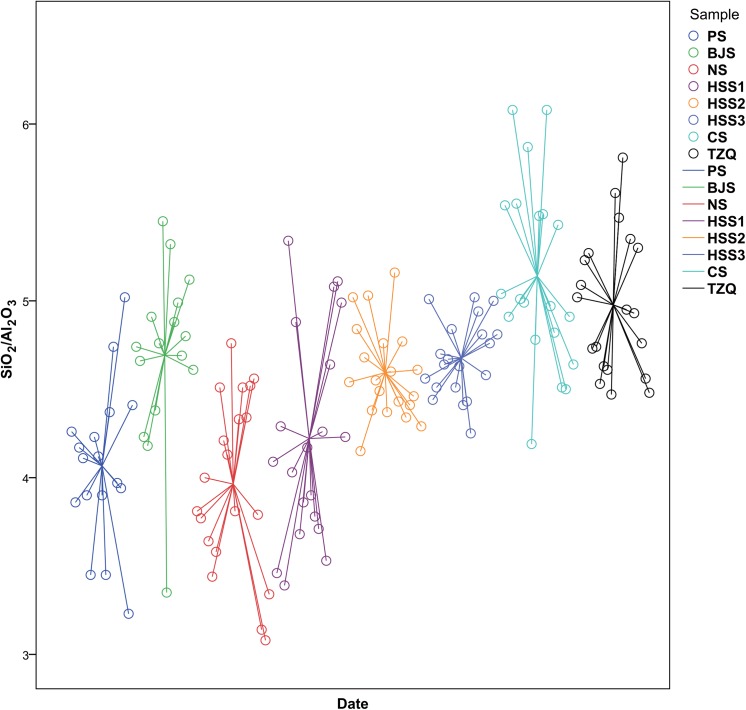
Date versus silica-alumina ratio in bodies of impressed stoneware and proto-porcelain (wt%). During Xia and Shang dynasties, the ratio was not stabilized, while the subsequent development witnessed a continuous rise from 4.2 to 5.1.

**Fig 6:**
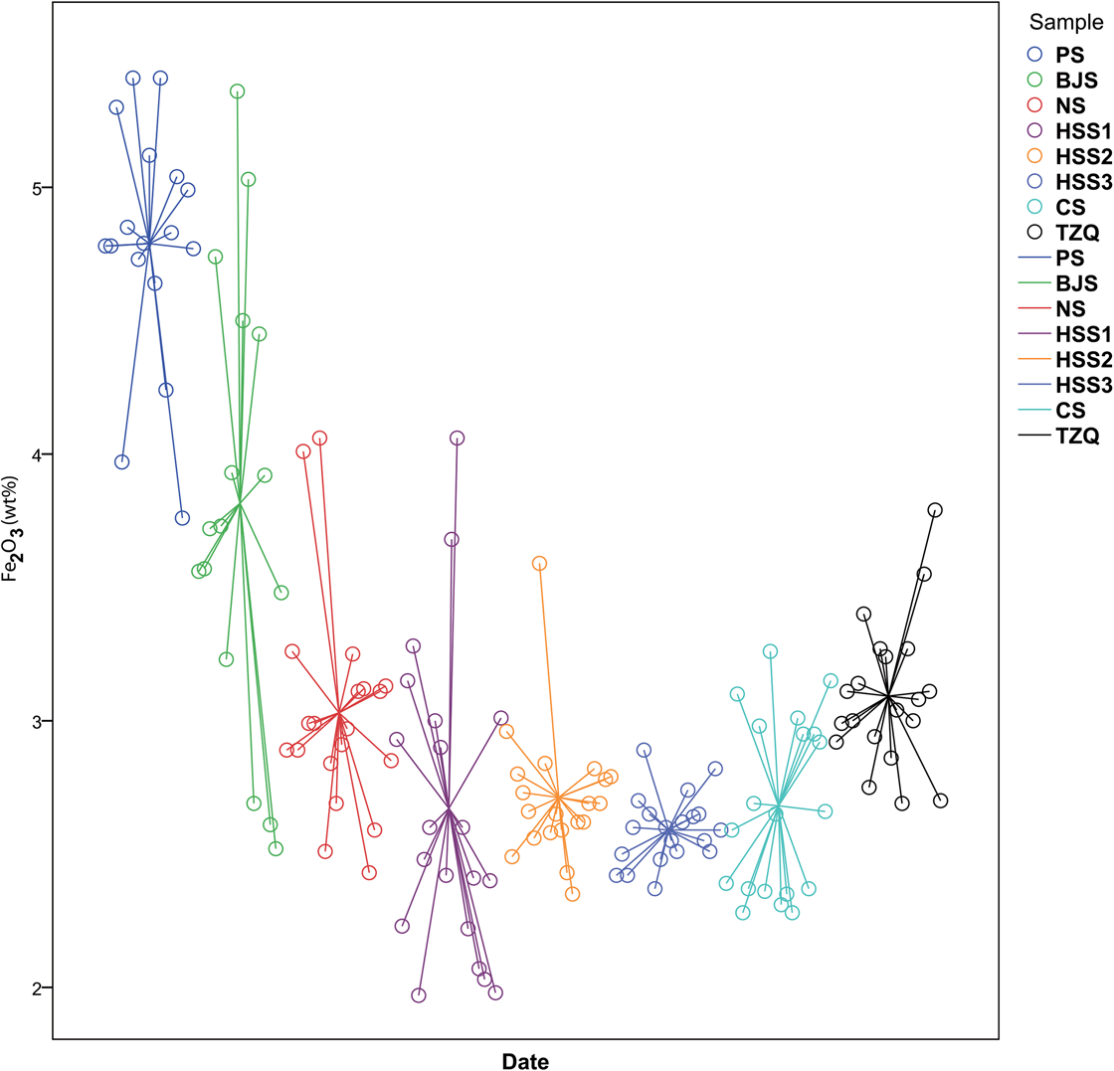
Date versus iron(III) oxide in bodies of impressed stoneware and proto-porcelain (wt%). During Xia and Shang dynasties, the iron(III) oxide level sharply decreased from 4.29 wt% to 2.53 wt%. Afterwards, it maintained a relatively stable level of 2–3 wt%.

According to principal components analysis, the raw materials of the bodies were procured locally. The contents of silicon dioxide and aluminium(III) oxide together account for over 90 wt% of the total composition, thus the two main components best characterised the raw materials. The silica-alumina ratio fluctuated between 3.9 and 5.1 from the Xia to the Warring States period, as shown in Fig 5. During the Xia and Shang dynasties, the ratio was not stabilised, while the subsequent development witnessed a continuous rise from 4.2 to 5.1. Meanwhile, a prominent trend in the chemical composition was the consecutive decline of the iron(III) oxide content. This contributed to higher whiteness of the bodies. The preferred formula composition was not completely established during the Xia and Shang dynasties, as the iron(III) oxide level sharply decreasing from 4.29 wt% to 2.53 wt%. In Fig 6, from the Shang to the Warring States period, the iron(III) oxide content maintained a relatively stable level of 2–3 wt%, during which only the Tingziqiao kiln used raw material with higher than average iron(III) oxide content (2.59 wt%). Afterwards, the iron(III) oxide content of typical ceramics in southern China remained about the same (2 wt%) for the next thousand years[41]. In addition, from the Xia to the Warring States period, for magnesium, phosphorus, calcium, titanium and manganese, only small fluctuations in the concentration of their oxides have been observed, suggesting that the geologic conditions of the sources of the raw material stayed almost unchanged for more than a thousand years. Given all that, it seemed that the ancients tried to look for better raw material from mineral resources with no significant differences. The appropriate formula composition was later derived through trial and error. Eventually, the persistent pursuit of optimal raw material led to the emergence of mature ceramics in East Han dynasty [49].

### Development of early glazes

It is known that raw materials for making glaze are generally porcelain stones and fluxes, such as calcium oxide and potassium oxide. Porcelain stones from different deposits vary somewhat in composition, and the uncertainties of plant ashes should be taken into account as well.

During the Xia, Shang and Zhou dynasties, almost all kilns were heated by firewood in southern China. Therefore, plant ashes were considered a material that could be easily found. Unlike limestone, plant ashes required no knapping or pulverizing to be prepared for glaze making, which made it highly accessible to early potters.

Used as firewood, the procured grass and wood might neither be from a single location nor be from plants of the same kind. Therefore, the outcome should consist of a mixture of multiple plant ashes. There has been a considerable amount of research on a variety of plant ashes with different chemical compositions. According to the literature statistics, the content of calcium oxide in woody plant ashes (10–70 wt%) is higher than that in herbaceous plant ashes (<10 wt%) [50][51].

**Fig 7:**
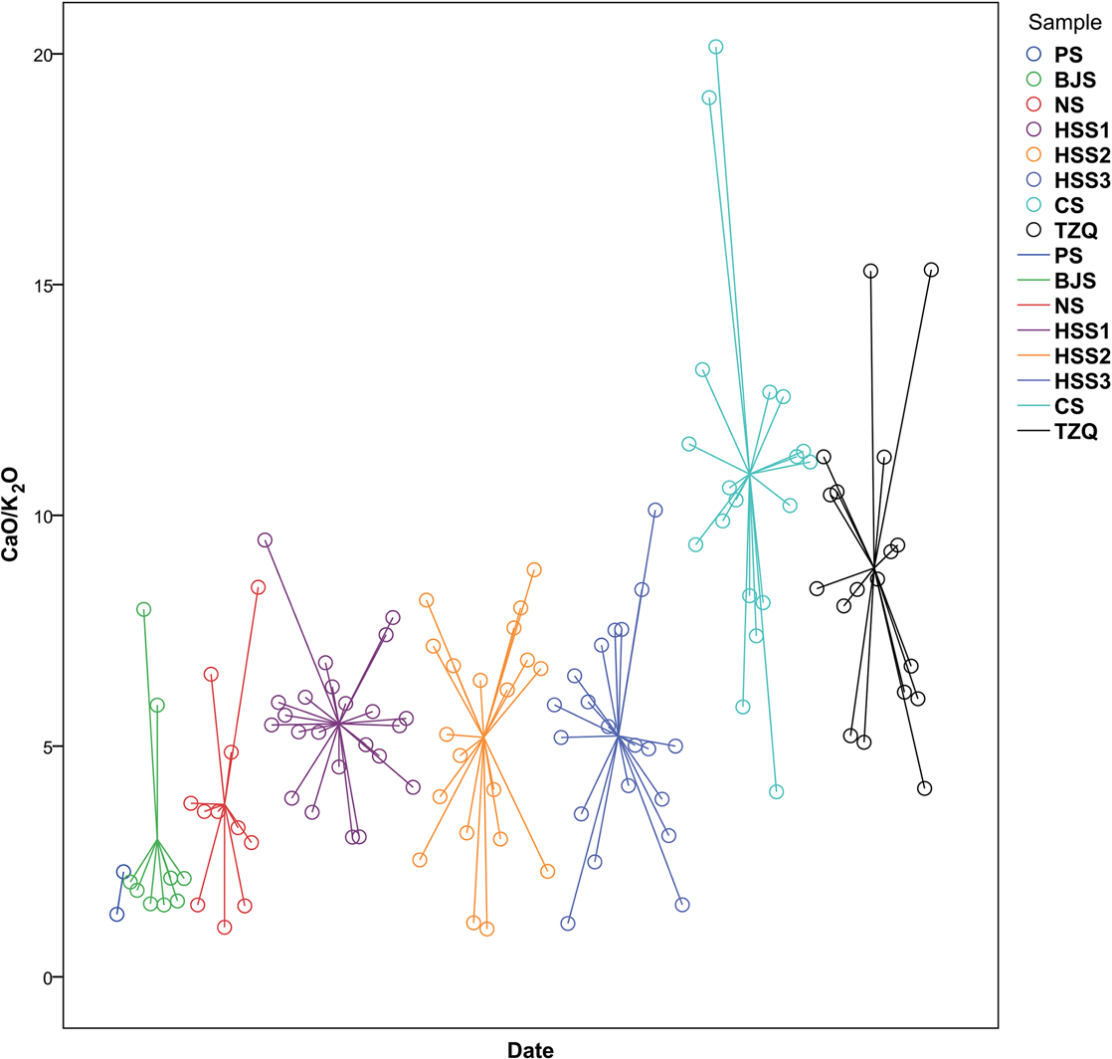
Date versus calcia-potash ratio in proto-porcelain glazes (wt%). The calcia-potash ratios of early Huzhou samples were less than 4 wt%. Huoshaoshan glazes maintained a level of around 5 wt% at three historical stages. Later a surge was seen in calcia-potash ratio, which nearly doubled during the Warring States period.

When the early potters applied the glaze mixture to the ceramic bodies, they might not know exactly what ingredients were in that formula. Based on long-term practical experience, potters gradually noticed that different plant ashes had different effects on formation of the glaze. The key factor for better forming the proto-porcelain glaze was an increased calcia-potash ratio, as shown in Fig 7. Specifically, fluxes rich in calcium could lead to a decreased melting temperature of the glaze. One single variety of plant ashes such as pine ashes and/or bamboo ashes was intentionally chosen therefore for the preparation of the glaze.

Considering the fact that potassium oxide dissolves in water easily, another factor that may come into play is, by elutriating raw materials of the glaze, the content of potassium oxide could be decreased.

Whether there was lime in the formula composition has not yet been proven. Some scholars hold a negative view of lime involved in the making of proto-porcelain glaze, arguing that the increase in phosphorus(V) oxide and magnesium oxide levels cannot be attributed to lime involvement [52][53]. Further studies are needed before we can know for sure whether lime was added to the formulation.

## Supporting Information

S1 Fig Photos of impressed stoneware and proto-porcelain sherds excavated from Piaoshan (PS) kiln site. Photograph: Huansheng Cheng.
(JPG)

S2 Fig Photos of impressed stoneware and proto-porcelain sherds excavated from Beijiashan (BJS) kiln site. Photograph: Huansheng Cheng.
(JPG)

S3 Fig Photos of proto-porcelain sherds excavated from Nanshan (NS) kiln site. Photograph: Huansheng Cheng.
(JPG)

S4 Fig The diagram of principal components analysis on proto-porcelain glazes from six kiln sites. The solid line ellipses represent the 95% confidence limit for samples from each kiln respectively.
(TIF)

S1 Table PIXE results of the chemical compositions (wt%) of the bodies of impressed stoneware and proto-porcelain sherds from 6 kiln sites. (DOC)

S2 Table PIXE results of the chemical compositions (wt%) of the surfaces of impressed stoneware and proto-porcelain sherds from 6 kiln sites. (DOC)

## References

[1] Wu X, Zhang C, Goldberg P, Cohen D, Pan Y, Arpin T, et al Early pottery at 20,000 years ago in Xianrendong Cave, China. Science. 2012 pp. 1696–1700. doi: 10.1126/science.1218643 22745428 10.1126/science.1218643

[2] Jiazhi L, Hongjie L, Liming G. Further study of the process of technological evolution of ancient Chinese pottery and porcelain. MRS Proceedings. 1992. p. 571.

[3] Li J. A History of Science and Technology in China-Ceramic Volume. Science Press, Beijing; 1998 (in Chinese).

[4] Mino Y. Brief Survey of Early Chinese Glazed Wares. Artibus Asiae. JSTOR; 1975;37: 39–52.

[5] An J. A discussion on Shang porcelain from Zhengzhou. Cult Reli. 1960; 68–70 (in Chinese).

[6] Luo H, Li J. The definition of proto-porcelain. Archaeology. 1998;7: 69–72 (in Chinese).

[7] Kerr R, Needham J, Wood N. Science and Civilisation in China: Volume 5, Chemistry and Chemical Technology, Part 12, Ceramic Technology. Cambridge University Press; 2004.

[8] Liu L, Chen X. The archaeology of China: from the late Paleolithic to the early Bronze Age Cambridge University Press; 2012.

[9] Thorp RL. Erlitou and the search for the Xia. Early China. JSTOR; 1991; 1–38.

[10] An J. Jinhuai An’s Collected Works on Archaeology. Zhongzhou Ancient Books Publishing House; 1999 (in Chinese).

[11] Zhu J. Research on the provenance of proto-porcelain produced in Shang and Zhou dynasties University of Science and Technology of China 2006 (in Chinese).

[12] Yuyun T. On the cultural exchange of south and north from the condition of the northern pro-porcelain unearthed. Cult Reli Cent China. 2012;1: 12–25 (in Chinese).

[13] Chang K. The archaeology of ancient China Yale University Press; 1986.

[14] Hongjie L, Jiazhi L, Liming G. Study on manufacture sites for the proto-porcelain excavated in northern China. J Chinese Ceram Soc. 1996;24: 297–302 (in Chinese).

[15] Tiemei C, Rapp G (Rip), Jing Z. INAA of Shang-Zhou period proto-porcelain and a discussion of related problems. Archaeology. 2003;7: 11 (in Chinese).

[16] Qijiang L, Maolin Z, Junming W, Juan W. Situation and prospect of science and technology research of proto-porcelain produced in the Shang and Zhou dynasty. China Ceram. 2012;48: 13–17 (in Chinese).

[17] Jianming Z, Yuanfu C, Yueming S, Yun C, Jianming Z, Youliang Y. proto-porcelain kilns of the Shang dynasty distributed in the middle reaches of East Tiaoxi River. Archaeology. 2011;7: 3–8 (in Chinese).

[18] Yuanfu C, Jianming Z, Jianzhong Z, Shengcheng F. A Brief Report of the Excavation of A Kiln Site of Warring States Period at Tingziqiao, Deqing. Cult Reli East. 2010;34: 6–19 (in Chinese).

[19] Jianming Z, Yuanfu C, Yueming S, Yun C, Sun X, Wang C, et al The excavation of the proto-porcelain kilns of the Shang dynasty at the Nanshan Hill in Huzhou City, Zhejiang. Archaeology. 2012;11: 4–15 (in Chinese).

[20] Qian H. Review of the symposium of the Exhibition of Archaeological Achievements of Zhejiang Proto-celadon and Huoshaoshan Kiln Site in Deqing. Palace Museum J. 2012;5: 151–158 (in Chinese).

[21] Yueming S, Jianming Z, Yuanfu C. “The Source of Porcelain” issue and the significant progress for the study of the origin of porcelain. China Cultural Relics News. 1 8 2014 (in Chinese).

[22] Jianming Z. A brief discussion on the decorative patterns of proto-porcelain of Shang and Zhou dynasties. Archaeology. 2012;11: 61–76 (in Chinese).

[23] Stöckli R, Vermote E, Saleous N, Simmon R, Herring D. The Blue Marble Next Generation-A true color earth dataset including seasonal dynamics from MODIS. Publ by NASA Earth Obs. 2005;

[24] Chen J-X, Li H-K, Ren C-G, Tang G-H, Wang X-D, Yang F-C, et al PIXE research with an external beam. Nucl Instruments Methods. Elsevier; 1980;168: 437–440.

[25] Cheng HS, Zhang ZQ, Zhang B, Yang FJ. Non-destructive analysis and identification of jade by PIXE. Nucl Instruments Methods Phys Res Sect B Beam Interact with Mater Atoms. Elsevier; 2004;219: 30–34.

[26] Zhang ZW, Gan FX, Cheng HS. PIXE analysis of nephrite minerals from different deposits. Nucl Instruments Methods Phys Res Sect B Beam Interact with Mater Atoms. Elsevier; 2011;269: 460–465.

[27] Zhang B, Cheng HS, Ma B, Li QH, Zhang P, Gan FX, et al PIXE and ICP-AES analysis of early glass unearthed from Xinjiang (China). Nucl Instruments Methods Phys Res Sect B Beam Interact with Mater Atoms. Elsevier; 2005;240: 559–564.

[28] Cheng HS, He WQ, Tang JY, Yang FJ, Wang JH. PIXE analysis of ancient Chinese Qing dynasty porcelain. Nucl Instruments Methods Phys Res Sect B Beam Interact with Mater Atoms. Elsevier; 1996;118: 377–381.

[29] Cheng HS, Zhang ZQ, Xia HN, Jiang JC, Yang FJ. Non-destructive analysis and appraisal of ancient Chinese porcelain by PIXE. Nucl Instruments Methods Phys Res Sect B Beam Interact with Mater Atoms. Elsevier; 2002;190: 488–491.

[30] Cheng HS, Zhang ZQ, Zhang B, Yang FJ. The non-destructive identification of early Chinese porcelain by PIXE. Nucl Instruments Methods Phys Res Sect B Beam Interact with Mater Atoms. Elsevier; 2004;219: 16–19.

[31] Govindaraju K. 1984 compilation of working values and sample description for 170 international reference samples of mainly silicate rocks and minerals. Geostand Newsl. Wiley Online Library; 1984;8: 3–16.

[32] Hall GEM, Pelchat J-C. Inductively coupled plasma emission spectrometric determination of boron and other oxo-anion forming elements in geological materials. Analyst. Royal Society of Chemistry; 1986;111: 1255–1260.

[33] Lin S, Peng C. Studies on the application of laser sampling-inductively coupled plasma atomic emission spectrometry to the determination of rare earth and refractory elements. J Anal At Spectrom. The Royal Society of Chemistry; 1990;5: 509–514.

[34] Campbell JL, Hopman TL, Maxwell JA, Nejedly Z. The Guelph PIXE software package III: alternative proton database. Nucl Instruments Methods Phys Res Sect B Beam Interact with Mater Atoms. Elsevier; 2000;170: 193–204.

[35] Wenquan H. Ion beam analysis and archaeometry Fudan University 1997 (in Chinese).

[36] Xianming F. History of Chinese Ceramics. Cultural Relics Publishing House; 1982 (in Chinese).

[37] Yanyi G. Raw materials for making porcelain and the characteristics of porcelain wares in north and south China in ancient times. Archaeometry. Wiley Online Library; 1987;29: 3–19.

[38] Juan W, Meng-Xuan H, Mao-Lin Z, Jun-Ming W, Qi-Jiang L, Lin W, et al EDXRF analysis of provenience characteristics of proto-porcelains from typical kiln sites in southern China. Chinese J Spectrosc Lab. 2012;29: 3284–3288 (in Chinese).

[39] Yap CT, Hua Y. Provenance study of famous Chinese greenware bodies using principal component analysis. Zeitschrift für Naturforschung A, J Phys Sci. 1994;49: 759–766.

[40] Wu J, Leung JPL, Li Z, Stokes MJ. EDXRF studies on Chinese Yue ware. X-Ray Spectrom. Wiley Online Library; 2002;31: 408–413.

[41] XIONG Y, GONG Y, XIA J, WU J. The analysis of porcelains from Yue kiln by EDXRF. Sci Conserv Archaeol. 2010;22: 28–34 (in Chinese).

[42] Yap C-T, Hua Y. Chinese greenware raw materials: principal component analysis of major and minor chemical constituents. Appl Spectrosc. Society for Applied Spectroscopy; 1995;49: 981–986.

[43] Luo HJ. Chinese Ancient Ceramics and Multivariate Statistical Analysis. Chinese Light Industry Publishing Company; 1997 (in Chinese).

[44] Chen T, Rapp G (Rip), Jing Z, He N. Provenance studies of the earliest Chinese protoporcelain using instrumental neutron activation analysis. J Archaeol Sci. Elsevier; 1999;26: 1003–1015.

[45] Bin Z, Huansheng C, Jianming Z. PIXE analysis of proto-porcelain excavated from the Huoshaoshan kiln site in Deqing County, Zhejiang Province. Nucl Tech. 2014;37: 050201 (in Chinese).

[46] Junming W, Maolin Z, Qijiang L, Juan W, Pengfei J, Mengxuan H. The technique features of proto-porcelain in the Warring States period unearthed from Deqing. China Ceram. 2011;47: 66–69 (in Chinese).

[47] Junming W, Maolin Z, Qijian L, Juan W, Lili W, Zhenglong Y. Causes of glaze crawling on proto-porcelain excavated from south China. J Ceram. 2011;32: 376–380 (in Chinese).

[48] Bin Z, Huansheng C, Jianming Z. PIXE analysis of proto-porcelain excavated from Tingziqiao kiln site of Deqing (China). Nucl Sci Tech. 2014;25: 30202.

[49] Medley M. The Chinese potter: a practical history of Chinese ceramics Phaidon Press; 1976.

[50] Guozhen L, Yanyi G. Technical foundation of famous Chinese porcelain Shanghai Scientific and Technical Publishers; 1988 (in Chinese).

[51] Tichane R. Ash glazes Krause Publications Craft; 1998.

[52] Yaocheng C, Xiaowei Z. Study on the proto-porcelain in Xia-Shang period and the origin of porcelain glaze. Proceedings of the International Symposium on Ancient Ceramics (ISAC 2002). 2002. pp. 32–40 (in Chinese).

[53] Wu J, Zhang M, Wu J, Li Q, Li J, Deng Z, et al Study on the diversification of origins and primary development of Chinese porcelain glazes. Sci China Technol Sci. Springer; 2011;54: 99–104.

