# Supplementary figures and images for: The Earliest Chinese Proto-Porcelain Excavated from Kiln Sites: An Elemental Analysis
Source: PLoS One. 2015 Nov 4;10(11):e0139970. doi: 10.1371/journal.pone.0139970 (PMC4633156; doi:10.1371/journal.pone.0139970)

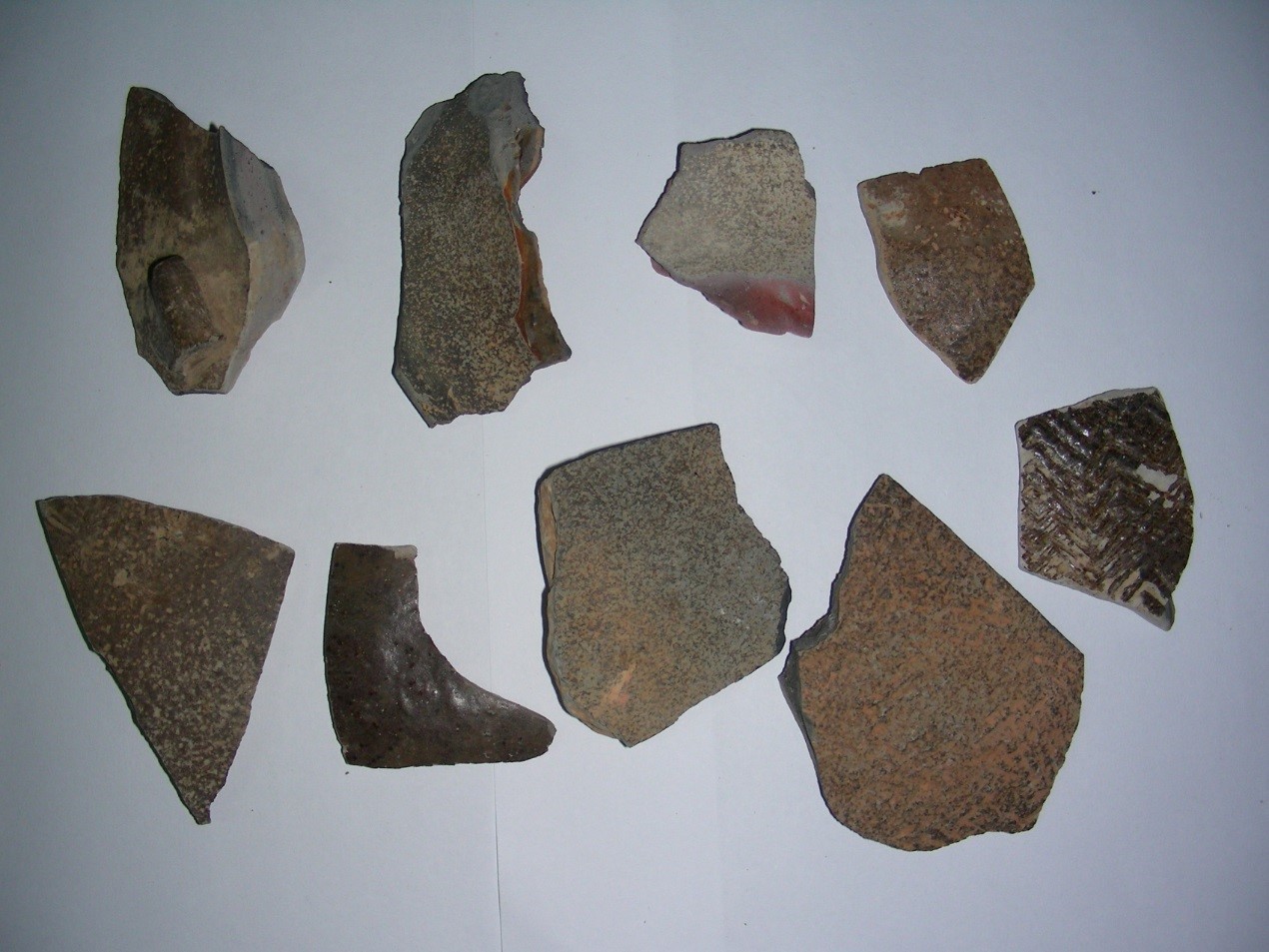

Supplement: S1 Fig — Photograph: Huansheng Cheng. (JPG) [file pone.0139970.s001.jpg]

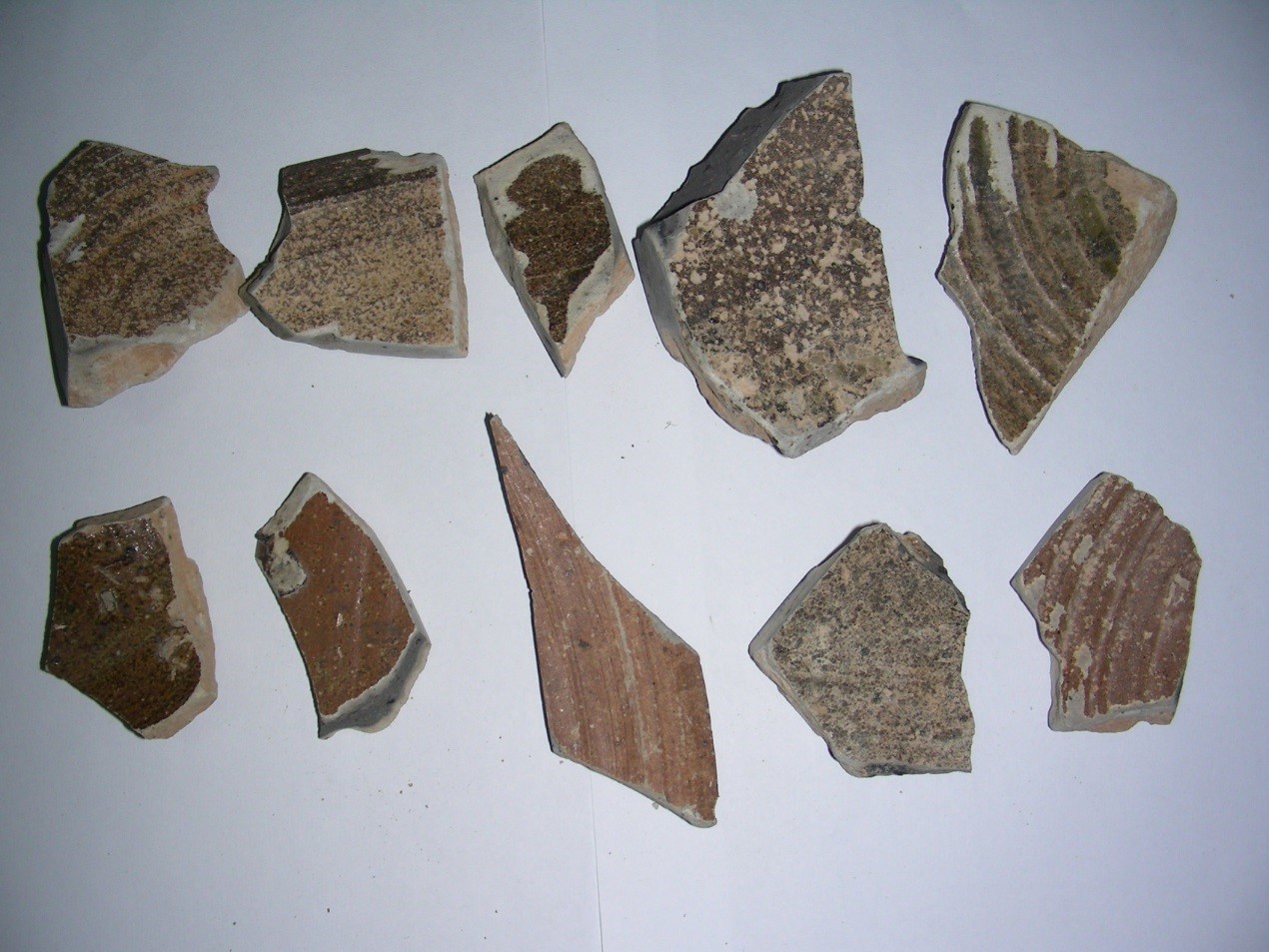

Supplement: S2 Fig — Photograph: Huansheng Cheng. (JPG) [file pone.0139970.s002.jpg]

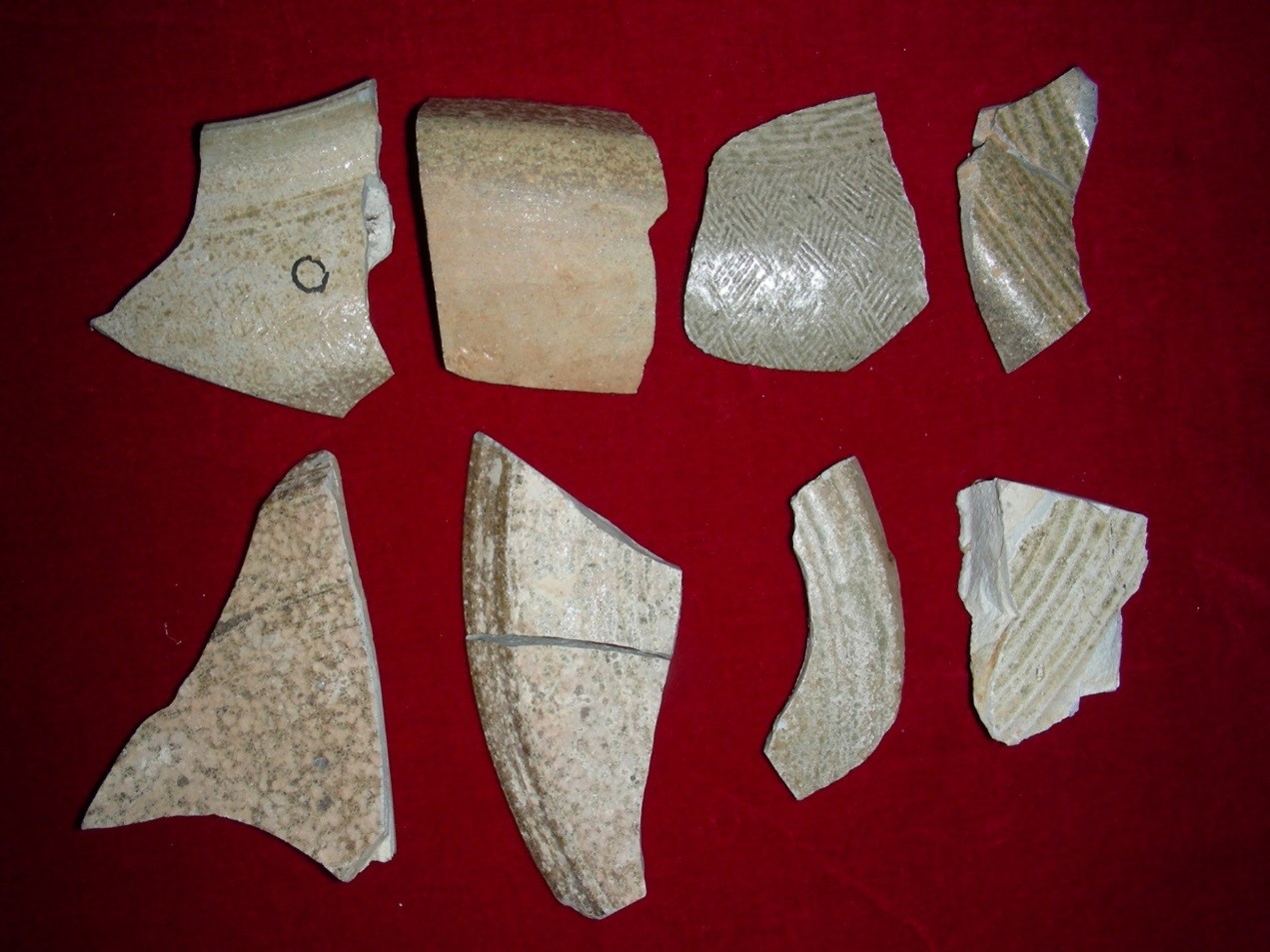

Supplement: S3 Fig — Photograph: Huansheng Cheng. (JPG) [file pone.0139970.s003.jpg]

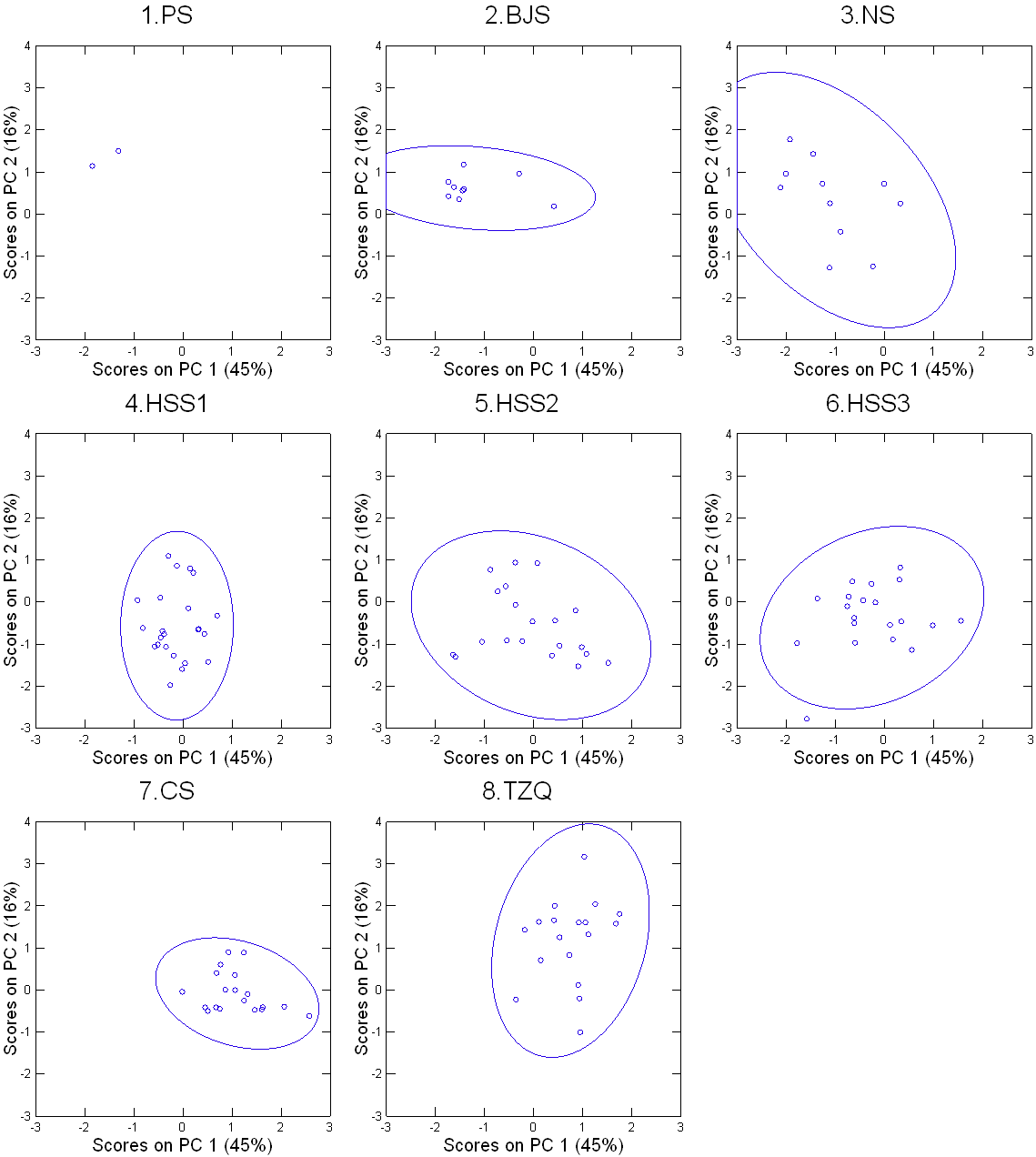

Supplement: S4 Fig — The solid line ellipses represent the 95% confidence limit for samples from each kiln respectively. (TIF) [file pone.0139970.s004.tif]
