# Supplementary material for: The Earliest Chinese Proto-Porcelain Excavated from Kiln Sites: An Elemental Analysis
Source: PLoS One. 2015 Nov 4;10(11):e0139970. doi: 10.1371/journal.pone.0139970 (PMC4633156; doi:10.1371/journal.pone.0139970)
Supplement: S1 Table — (DOC) [file pone.0139970.s005.doc]

**S1 Table. PIXE results of the chemical compositions (wt%) of the bodies of impressed stoneware and proto-porcelain sherds from 6 kiln sites.**

| No. | Data number | Classification | Na_2_O | MgO | Al_2_O_3_ | SiO_2_ | P_2_O_5_ | K_2_O | CaO | TiO_2_ | MnO | Fe_2_O_3_ | Total | Kiln |
| --- | --- | --- | --- | --- | --- | --- | --- | --- | --- | --- | --- | --- | --- | --- |
| 1 | HPT45:1-B | impressed stoneware | 0.24 | 0.50 | 17.33 | 73.82 | 0.10 | 2.14 | 0.36 | 1.05 | 0.03 | 4.28 | 99.85 | PS |
| 2 | HPT45:2-B | impressed stoneware | 0.33 | 0.97 | 18.60 | 71.87 | 0.15 | 2.31 | 0.33 | 1.08 | 0.04 | 4.28 | 99.96 | PS |
| 3 | HPT45:3-B | proto-porcelain | 0.29 | 0.80 | 17.48 | 72.82 | 0.18 | 2.04 | 0.36 | 1.08 | 0.03 | 4.80 | 99.88 | PS |
| 4 | HPT45:4-B | impressed stoneware | 0.31 | 0.61 | 17.97 | 73.86 | 0.12 | 2.10 | 0.31 | 1.11 | 0.03 | 3.47 | 99.89 | PS |
| 5 | HPT45:5-B | impressed stoneware | 0.35 | 0.89 | 18.51 | 72.11 | 0.08 | 2.28 | 0.35 | 1.04 | 0.02 | 4.35 | 99.98 | PS |
| 6 | HPT45:6-B | impressed stoneware | 0.18 | 0.75 | 20.40 | 70.34 | 0.07 | 1.90 | 0.30 | 1.03 | 0.03 | 4.91 | 99.91 | PS |
| 7 | HPT45:7-B | impressed stoneware | 0.21 | 0.71 | 17.45 | 73.77 | 0.15 | 2.02 | 0.37 | 1.04 | 0.03 | 4.23 | 99.98 | PS |
| 8 | HPT45:8-B | impressed stoneware | 0.30 | 0.69 | 17.74 | 73.15 | 0.18 | 2.07 | 0.36 | 1.11 | 0.03 | 4.29 | 99.92 | PS |
| 9 | HPT47:1-B | impressed stoneware | 0.27 | 0.68 | 18.57 | 72.44 | 0.05 | 1.87 | 0.29 | 1.10 | 0.05 | 4.62 | 99.94 | PS |
| 10 | HPT47:2-B | impressed stoneware | 0.26 | 0.62 | 20.43 | 70.54 | 0.11 | 2.26 | 0.46 | 0.99 | 0.04 | 4.14 | 99.85 | PS |
| 11 | HPT47:3-B | impressed stoneware | 0.28 | 0.62 | 16.95 | 74.06 | 0.16 | 1.71 | 0.21 | 1.00 | 0.02 | 4.91 | 99.92 | PS |
| 12 | HPT47:4-B | proto-porcelain | 0.11 | 0.47 | 16.06 | 76.05 | 0.16 | 1.93 | 0.27 | 1.11 | 0.03 | 3.74 | 99.93 | PS |
| 13 | HPT47:5-B | impressed stoneware | 0.29 | 0.83 | 18.26 | 72.55 | 0.20 | 2.16 | 0.30 | 1.02 | 0.03 | 4.33 | 99.97 | PS |
| 14 | HPT47:6-B | impressed stoneware | 0.15 | 0.71 | 18.45 | 72.78 | 0.12 | 1.75 | 0.25 | 1.15 | 0.03 | 4.54 | 99.93 | PS |
| 15 | HPT47:7-B | impressed stoneware | 0.11 | 0.43 | 15.46 | 77.66 | 0.17 | 1.42 | 0.10 | 1.18 | 0.09 | 3.26 | 99.88 | PS |
| 16 | HPT47:8-B | impressed stoneware | 0.19 | 0.62 | 21.52 | 69.59 | 0.07 | 1.79 | 0.31 | 1.36 | 0.02 | 4.49 | 99.96 | PS |
| 17 | HPT47:9-B | impressed stoneware | 0.33 | 0.70 | 16.85 | 74.28 | 0.24 | 1.90 | 0.23 | 1.06 | 0.03 | 4.27 | 99.89 | PS |
| 18 | HBT13:1-B | impressed stoneware | 0.71 | 0.64 | 16.07 | 76.10 | 0.18 | 1.86 | 0.44 | 0.83 | 0.03 | 3.06 | 99.92 | BJS |
| 19 | HBT13:2-B | proto-porcelain | 0.75 | 0.62 | 16.24 | 75.60 | 0.27 | 2.04 | 0.41 | 0.90 | 0.03 | 3.07 | 99.93 | BJS |
| 20 | HBT13:3-B | proto-porcelain | 0.66 | 0.54 | 17.62 | 74.51 | 0.33 | 1.75 | 0.31 | 0.99 | 0.02 | 3.22 | 99.95 | BJS |
| 21 | HBT13:4-B | impressed stoneware | 0.24 | 0.84 | 17.60 | 73.48 | 0.21 | 2.00 | 0.33 | 1.01 | 0.05 | 4.24 | 100.00 | BJS |
| 22 | HBT13:5-B | proto-porcelain | 0.59 | 0.53 | 15.42 | 75.65 | 0.93 | 2.12 | 0.46 | 0.98 | 0.01 | 3.23 | 99.92 | BJS |
| 23 | HBT13:6-B | impressed stoneware | 0.58 | 0.66 | 17.16 | 75.18 | 0.20 | 2.04 | 0.46 | 0.89 | 0.01 | 2.73 | 99.91 | BJS |
| 24 | HBT13:7-B | proto-porcelain | 0.46 | 0.53 | 15.87 | 75.56 | 0.98 | 1.58 | 0.39 | 1.15 | 0.01 | 3.43 | 99.96 | BJS |
| 25 | HBT13:8-B | impressed stoneware | 0.23 | 0.60 | 14.09 | 76.73 | 0.40 | 1.58 | 0.24 | 1.14 | 0.06 | 4.86 | 99.93 | BJS |
| 26 | HBT13:9-B | proto-porcelain | 0.35 | 0.58 | 20.95 | 70.27 | 0.61 | 1.71 | 0.33 | 1.14 | 0.02 | 4.00 | 99.96 | BJS |
| 27 | HBT13:10-B | impressed stoneware | 0.36 | 0.70 | 14.41 | 76.63 | 0.21 | 1.74 | 0.28 | 1.05 | 0.06 | 4.53 | 99.97 | BJS |
| 28 | HBT13:15-B | proto-porcelain | 0.81 | 0.56 | 15.79 | 76.99 | 0.27 | 2.06 | 0.46 | 0.85 | 0.01 | 2.19 | 99.99 | BJS |
| 29 | HBT13:16-B | impressed stoneware | 0.68 | 0.65 | 15.19 | 75.83 | 0.23 | 2.07 | 0.46 | 0.90 | 0.03 | 3.95 | 99.99 | BJS |
| 30 | HBT13:17-B | proto-porcelain | 0.80 | 0.66 | 16.08 | 75.34 | 0.37 | 1.98 | 0.39 | 0.95 | 0.01 | 3.42 | 100.00 | BJS |
| 31 | HBT13:18-B | impressed stoneware | 0.65 | 0.47 | 16.09 | 77.29 | 0.23 | 1.90 | 0.32 | 0.81 | 0.03 | 2.11 | 99.90 | BJS |
| 32 | HBT13:19-B | impressed stoneware | 0.95 | 0.63 | 15.24 | 78.02 | 0.11 | 1.73 | 0.34 | 0.86 | 0.01 | 2.02 | 99.91 | BJS |
| 33 | HBT13:20-B | proto-porcelain | 0.74 | 0.62 | 16.41 | 75.63 | 0.26 | 2.07 | 0.39 | 0.86 | 0.01 | 2.98 | 99.97 | BJS |
| 34 | HNG1:1-B | proto-porcelain | 0.83 | 0.59 | 19.10 | 72.79 | 0.23 | 2.63 | 0.45 | 0.95 | 0.01 | 2.39 | 99.97 | NS |
| 35 | HNG1:2-B | impressed stoneware | 0.65 | 0.52 | 19.24 | 72.48 | 0.28 | 2.60 | 0.35 | 1.03 | 0.04 | 2.76 | 99.95 | NS |
| 36 | HNG1:3-B | impressed stoneware | 0.97 | 0.44 | 18.29 | 73.24 | 0.17 | 2.89 | 0.43 | 0.94 | 0.01 | 2.39 | 99.77 | NS |
| 37 | HNG1:4-B | proto-porcelain | 0.83 | 0.56 | 19.58 | 71.30 | 0.14 | 2.15 | 0.85 | 0.96 | 0.06 | 3.51 | 99.94 | NS |
| 38 | HNT4022:1-B | proto-porcelain | 0.86 | 0.57 | 20.69 | 71.27 | 0.35 | 2.27 | 0.43 | 0.91 | 0.01 | 2.49 | 99.85 | NS |
| 39 | HNT4022:2-B | impressed stoneware | 1.14 | 0.62 | 20.12 | 72.02 | 0.14 | 2.19 | 0.37 | 0.86 | 0.03 | 2.49 | 99.98 | NS |
| 40 | HNT4028:1-B | impressed stoneware | 1.43 | 0.66 | 16.05 | 72.43 | 0.27 | 3.81 | 0.74 | 0.88 | 0.06 | 3.56 | 99.89 | NS |
| 41 | HNT4028:2-B | impressed stoneware | 1.34 | 0.53 | 17.75 | 74.76 | 0.19 | 2.18 | 0.25 | 0.87 | 0.02 | 2.01 | 99.90 | NS |
| 42 | HNT4042:1-B | proto-porcelain | 1.27 | 0.42 | 17.90 | 73.99 | 0.42 | 2.31 | 0.31 | 0.92 | 0.04 | 2.34 | 99.92 | NS |
| 43 | HNT4042:2-B | impressed stoneware | 0.91 | 0.44 | 15.96 | 75.99 | 0.10 | 2.86 | 0.40 | 0.98 | 0.03 | 2.19 | 99.86 | NS |
| 44 | HNT4043:1-B | proto-porcelain | 0.84 | 0.57 | 19.14 | 72.89 | 0.26 | 2.55 | 0.33 | 0.97 | 0.01 | 2.41 | 99.97 | NS |
| 45 | HNT4044:1-B | proto-porcelain | 1.06 | 0.65 | 17.22 | 74.64 | 0.05 | 2.66 | 0.32 | 0.84 | 0.03 | 2.47 | 99.94 | NS |
| 46 | HNT4045:1-B | proto-porcelain | 0.83 | 0.39 | 16.61 | 74.90 | 0.21 | 2.95 | 0.31 | 0.97 | 0.03 | 2.75 | 99.95 | NS |
| 47 | HNT4045:2-B | impressed stoneware | 1.00 | 0.55 | 17.10 | 74.29 | 0.35 | 2.67 | 0.38 | 1.00 | 0.04 | 2.61 | 99.99 | NS |
| 48 | HNT4046:1-B | impressed stoneware | 1.07 | 0.43 | 16.54 | 74.84 | 0.24 | 2.75 | 0.41 | 0.95 | 0.02 | 2.62 | 99.87 | NS |
| 49 | HNT4046:2-B | proto-porcelain | 1.30 | 0.43 | 16.59 | 75.66 | 0.33 | 2.44 | 0.35 | 0.93 | 0.02 | 1.93 | 99.98 | NS |
| 50 | HNT4047:1-B | impressed stoneware | 1.03 | 0.58 | 19.27 | 73.06 | 0.43 | 2.33 | 0.35 | 0.81 | 0.03 | 2.09 | 99.98 | NS |
| 51 | HNT4047:2-B | proto-porcelain | 0.86 | 0.71 | 22.25 | 69.80 | 0.31 | 2.11 | 0.39 | 0.92 | 0.03 | 2.61 | 99.99 | NS |
| 52 | HNT4047:3-B | proto-porcelain | 1.00 | 0.64 | 22.53 | 69.49 | 0.40 | 2.07 | 0.35 | 0.84 | 0.01 | 2.63 | 99.96 | NS |
| 53 | HNT4047:4-B | proto-porcelain | 1.43 | 0.52 | 21.26 | 70.91 | 0.36 | 1.94 | 0.34 | 0.89 | 0.00 | 2.35 | 100.00 | NS |
| 54 | DHT5045:1-B | proto-porcelain | 0.62 | 0.60 | 18.29 | 74.89 | 0.52 | 1.65 | 0.14 | 0.82 | 0.00 | 2.43 | 99.96 | HSS1 |
| 55 | DHT5045:2-B | proto-porcelain | 0.33 | 0.72 | 20.94 | 72.36 | 0.29 | 2.40 | 0.28 | 0.84 | 0.02 | 1.73 | 99.91 | HSS1 |
| 56 | DHT5045:3-B | proto-porcelain | 0.43 | 0.55 | 17.54 | 75.18 | 0.16 | 2.05 | 0.42 | 0.89 | 0.02 | 2.65 | 99.89 | HSS1 |
| 57 | DHT5045:4-B | proto-porcelain | 0.19 | 0.73 | 20.95 | 70.92 | 0.17 | 2.93 | 0.29 | 1.02 | 0.00 | 2.78 | 99.98 | HSS1 |
| 58 | DHT5045:5-B | proto-porcelain | 0.44 | 0.34 | 14.82 | 79.11 | 0.33 | 2.00 | 0.24 | 1.03 | 0.01 | 1.47 | 99.79 | HSS1 |
| 59 | DHT5045:6-B | proto-porcelain | 0.32 | 0.69 | 18.46 | 74.40 | 0.27 | 2.48 | 0.30 | 0.92 | 0.01 | 1.98 | 99.83 | HSS1 |
| 60 | DHT5045:7-B | proto-porcelain | 0.73 | 0.45 | 15.97 | 77.89 | 0.41 | 1.14 | 0.25 | 0.91 | 0.04 | 2.10 | 99.89 | HSS1 |
| 61 | DHT5045:8-B | proto-porcelain | 0.26 | 0.43 | 20.02 | 73.63 | 0.14 | 1.54 | 0.19 | 1.22 | 0.02 | 2.50 | 99.95 | HSS1 |
| 62 | DHT5045:9-B | proto-porcelain | 0.37 | 0.61 | 19.16 | 73.92 | 0.32 | 1.88 | 0.16 | 1.03 | 0.02 | 2.40 | 99.87 | HSS1 |
| 63 | DHT5045:10-B | proto-porcelain | 0.37 | 0.58 | 18.07 | 75.38 | 0.14 | 2.27 | 0.29 | 0.85 | 0.01 | 1.92 | 99.88 | HSS1 |
| 64 | DHT5045:11-B | proto-porcelain | 0.43 | 0.67 | 18.86 | 73.53 | 0.21 | 1.76 | 0.30 | 0.92 | 0.02 | 3.18 | 99.88 | HSS1 |
| 65 | DHT5045:12-B | proto-porcelain | 0.30 | 0.68 | 19.36 | 73.11 | 0.22 | 1.54 | 0.19 | 1.00 | 0.02 | 3.56 | 99.98 | HSS1 |
| 66 | DHT5045:13-B | proto-porcelain | 0.29 | 0.60 | 19.82 | 73.57 | 0.37 | 2.07 | 0.24 | 0.84 | 0.02 | 2.10 | 99.92 | HSS1 |
| 67 | DHT5045:14-B | proto-porcelain | 0.65 | 0.44 | 17.35 | 73.99 | 0.39 | 3.65 | 0.77 | 1.03 | 0.01 | 1.72 | 100.00 | HSS1 |
| 68 | DHT5045:15-B | proto-porcelain | 0.56 | 0.62 | 20.60 | 72.63 | 0.00 | 2.66 | 0.29 | 0.64 | 0.02 | 1.91 | 99.93 | HSS1 |
| 69 | DHT5045:16-B | proto-porcelain | 0.26 | 0.55 | 16.66 | 77.27 | 0.48 | 2.00 | 0.23 | 0.95 | 0.01 | 1.57 | 99.98 | HSS1 |
| 70 | DHT5045:17-B | proto-porcelain | 0.48 | 0.49 | 15.45 | 78.48 | 0.14 | 2.18 | 0.24 | 1.00 | 0.01 | 1.53 | 100.00 | HSS1 |
| 71 | DHT5045:18-B | proto-porcelain | 0.30 | 0.51 | 15.44 | 78.87 | 0.14 | 1.73 | 0.17 | 0.93 | 0.01 | 1.90 | 100.00 | HSS1 |
| 72 | DHT5045:19-B | proto-porcelain | 0.45 | 0.53 | 15.65 | 78.12 | 0.39 | 2.09 | 0.24 | 0.93 | 0.01 | 1.48 | 99.89 | HSS1 |
| 73 | DHT5045:20-B | proto-porcelain | 0.47 | 0.57 | 17.80 | 75.33 | 0.34 | 1.82 | 0.16 | 0.97 | 0.01 | 2.51 | 99.98 | HSS1 |
| 74 | DHIIT3036:1-B | proto-porcelain | 0.69 | 0.47 | 16.67 | 75.60 | 0.05 | 2.65 | 0.40 | 0.91 | 0.04 | 2.46 | 99.94 | HSS2 |
| 75 | DHIIT3036:2-B | proto-porcelain | 0.62 | 0.42 | 15.42 | 77.39 | 0.15 | 2.68 | 0.34 | 0.91 | 0.00 | 1.99 | 99.92 | HSS2 |
| 76 | DHIIT3036:3-B | proto-porcelain | 0.59 | 0.49 | 15.94 | 77.12 | 0.12 | 2.07 | 0.38 | 0.91 | 0.03 | 2.30 | 99.95 | HSS2 |
| 77 | DHIIT3036:4-B | proto-porcelain | 0.61 | 0.58 | 18.01 | 74.80 | 0.14 | 2.34 | 0.39 | 0.88 | 0.02 | 2.23 | 100.00 | HSS2 |
| 78 | DHIIT3036:5-B | proto-porcelain | 0.56 | 0.42 | 16.37 | 76.66 | 0.05 | 2.47 | 0.37 | 0.85 | 0.03 | 2.16 | 99.94 | HSS2 |
| 79 | DHIIT3036:6-B | proto-porcelain | 0.70 | 0.50 | 15.41 | 77.56 | 0.09 | 2.29 | 0.33 | 0.95 | 0.02 | 2.06 | 99.91 | HSS2 |
| 80 | DHIIT3036:7-B | proto-porcelain | 0.55 | 0.54 | 17.17 | 75.18 | 0.12 | 2.06 | 0.38 | 0.86 | 0.02 | 3.09 | 99.97 | HSS2 |
| 81 | DHIIT3036:8-B | proto-porcelain | 0.57 | 0.51 | 16.72 | 76.01 | 0.15 | 2.42 | 0.39 | 0.83 | 0.04 | 2.34 | 99.98 | HSS2 |
| 82 | DHIIT3036:9-B | proto-porcelain | 0.63 | 0.51 | 16.95 | 76.15 | 0.14 | 2.28 | 0.36 | 0.83 | 0.04 | 2.08 | 99.97 | HSS2 |
| 83 | DHIIT3036:10-B | proto-porcelain | 0.62 | 0.58 | 16.13 | 76.78 | 0.12 | 2.23 | 0.42 | 0.91 | 0.04 | 2.15 | 99.98 | HSS2 |
| 84 | DHIIT3036:11-B | proto-porcelain | 0.66 | 0.55 | 17.32 | 75.69 | 0.13 | 2.16 | 0.40 | 0.86 | 0.01 | 2.09 | 99.87 | HSS2 |
| 85 | DHIIT3036:12-B | proto-porcelain | 0.67 | 0.41 | 16.59 | 76.39 | 0.15 | 2.59 | 0.33 | 0.83 | 0.03 | 1.93 | 99.92 | HSS2 |
| 86 | DHIIT3036:13-B | proto-porcelain | 0.65 | 0.45 | 15.16 | 78.26 | 0.09 | 2.24 | 0.32 | 0.92 | 0.03 | 1.85 | 99.97 | HSS2 |
| 87 | DHIIT3036:14-B | proto-porcelain | 0.74 | 0.42 | 17.10 | 75.72 | 0.15 | 2.57 | 0.28 | 0.86 | 0.01 | 2.12 | 99.97 | HSS2 |
| 88 | DHIIT3036:15-B | proto-porcelain | 0.72 | 0.42 | 16.07 | 76.58 | 0.05 | 2.79 | 0.30 | 0.92 | 0.02 | 2.12 | 99.99 | HSS2 |
| 89 | DHIIT3036:16-B | proto-porcelain | 0.63 | 0.46 | 17.33 | 75.20 | 0.26 | 2.67 | 0.34 | 0.88 | 0.01 | 2.19 | 99.97 | HSS2 |
| 90 | DHIIT3036:17-B | proto-porcelain | 0.56 | 0.49 | 17.14 | 75.61 | 0.10 | 2.40 | 0.42 | 0.92 | 0.03 | 2.32 | 99.99 | HSS2 |
| 91 | DHIIT3036:18-B | proto-porcelain | 0.64 | 0.59 | 17.04 | 76.07 | 0.08 | 2.21 | 0.36 | 0.80 | 0.02 | 2.19 | 100.00 | HSS2 |
| 92 | DHIIT3036:19-B | proto-porcelain | 0.71 | 0.53 | 16.53 | 76.25 | 0.05 | 2.25 | 0.34 | 0.99 | 0.01 | 2.28 | 99.94 | HSS2 |
| 93 | DHIIT3036:20-B | proto-porcelain | 0.57 | 0.48 | 17.55 | 75.28 | 0.05 | 2.47 | 0.35 | 0.86 | 0.01 | 2.29 | 99.91 | HSS2 |
| 94 | DHIIIT3032:1-B | proto-porcelain | 0.90 | 0.46 | 16.66 | 75.90 | 0.04 | 2.78 | 0.36 | 0.85 | 0.04 | 1.92 | 99.91 | HSS3 |
| 95 | DHIIIT3032:2-B | proto-porcelain | 0.77 | 0.40 | 15.37 | 77.08 | 0.22 | 2.71 | 0.39 | 1.03 | 0.04 | 2.00 | 100.01 | HSS3 |
| 96 | DHIIIT3032:3-B | proto-porcelain | 0.94 | 0.47 | 16.91 | 75.02 | 0.25 | 2.98 | 0.38 | 1.03 | 0.02 | 1.92 | 99.92 | HSS3 |
| 97 | DHIIIT3032:4-B | proto-porcelain | 0.76 | 0.40 | 16.79 | 75.66 | 0.14 | 2.70 | 0.35 | 1.04 | 0.04 | 2.10 | 99.98 | HSS3 |
| 98 | DHIIIT3032:5-B | proto-porcelain | 0.79 | 0.35 | 16.15 | 75.94 | 0.05 | 3.15 | 0.35 | 1.00 | 0.02 | 2.20 | 100.00 | HSS3 |
| 99 | DHIIIT3032:6-B | proto-porcelain | 0.79 | 0.49 | 16.32 | 75.79 | 0.06 | 2.78 | 0.36 | 0.96 | 0.02 | 2.39 | 99.96 | HSS3 |
| 100 | DHIIIT3032:7-B | proto-porcelain | 0.81 | 0.39 | 16.23 | 75.82 | 0.14 | 2.99 | 0.44 | 0.95 | 0.03 | 2.15 | 99.95 | HSS3 |
| 101 | DHIIIT3032:8-B | proto-porcelain | 0.87 | 0.39 | 15.84 | 76.74 | 0.24 | 2.61 | 0.36 | 0.90 | 0.03 | 1.87 | 99.85 | HSS3 |
| 102 | DHIIIT3032:9-B | proto-porcelain | 0.70 | 0.44 | 16.77 | 75.65 | 0.25 | 2.82 | 0.37 | 0.98 | 0.04 | 1.98 | 100.00 | HSS3 |
| 103 | DHIIIT3032:10-B | proto-porcelain | 0.77 | 0.54 | 16.32 | 75.61 | 0.22 | 2.95 | 0.46 | 0.95 | 0.01 | 2.10 | 99.93 | HSS3 |
| 104 | DHIIIT3032:11-B | proto-porcelain | 0.91 | 0.41 | 17.03 | 75.08 | 0.28 | 2.79 | 0.39 | 0.96 | 0.03 | 2.05 | 99.93 | HSS3 |
| 105 | DHIIIT3032:12-B | proto-porcelain | 0.87 | 0.39 | 17.04 | 75.53 | 0.08 | 2.75 | 0.36 | 0.93 | 0.03 | 2.01 | 99.99 | HSS3 |
| 106 | DHIIIT3032:13-B | proto-porcelain | 0.80 | 0.40 | 17.63 | 74.85 | 0.22 | 2.62 | 0.42 | 0.91 | 0.03 | 2.12 | 100.00 | HSS3 |
| 107 | DHIIIT3032:14-B | proto-porcelain | 0.86 | 0.45 | 15.28 | 76.76 | 0.17 | 2.87 | 0.34 | 0.95 | 0.03 | 2.24 | 99.95 | HSS3 |
| 108 | DHIIIT3032:15-B | proto-porcelain | 0.73 | 0.47 | 15.61 | 77.08 | 0.09 | 2.35 | 0.40 | 1.03 | 0.03 | 2.14 | 99.93 | HSS3 |
| 109 | DHIIIT3032:16-B | proto-porcelain | 0.69 | 0.49 | 15.85 | 76.31 | 0.22 | 2.84 | 0.34 | 1.04 | 0.02 | 2.15 | 99.95 | HSS3 |
| 110 | DHIIIT3032:17-B | proto-porcelain | 1.00 | 0.41 | 16.44 | 75.23 | 0.00 | 3.29 | 0.38 | 0.99 | 0.03 | 2.05 | 99.82 | HSS3 |
| 111 | DHIIIT3032:18-B | proto-porcelain | 0.79 | 0.49 | 16.02 | 76.31 | 0.06 | 2.86 | 0.44 | 0.91 | 0.02 | 2.01 | 99.91 | HSS3 |
| 112 | DHIIIT3032:19-B | proto-porcelain | 0.70 | 0.46 | 15.40 | 76.93 | 0.24 | 2.54 | 0.39 | 0.98 | 0.04 | 2.32 | 100.00 | HSS3 |
| 113 | DHIIIT3032:20-B | proto-porcelain | 0.93 | 0.31 | 15.82 | 76.15 | 0.06 | 2.93 | 0.44 | 1.05 | 0.03 | 2.09 | 99.81 | HSS3 |
| 114 | DCT12:1-B | proto-porcelain | 0.61 | 0.59 | 15.45 | 77.88 | 0.22 | 1.87 | 0.39 | 1.08 | 0.02 | 1.89 | 100.00 | CS |
| 115 | DCT12:2-B | proto-porcelain | 0.58 | 0.52 | 14.31 | 79.33 | 0.21 | 1.49 | 0.36 | 1.05 | 0.01 | 2.09 | 99.95 | CS |
| 116 | DCT12:3-B | proto-porcelain | 0.68 | 0.52 | 15.69 | 76.96 | 0.05 | 1.91 | 0.45 | 1.08 | 0.03 | 2.60 | 99.97 | CS |
| 117 | DCT12:4-B | proto-porcelain | 0.69 | 0.37 | 13.26 | 80.61 | 0.16 | 1.70 | 0.33 | 1.04 | 0.02 | 1.78 | 99.96 | CS |
| 118 | DCT12:5-B | proto-porcelain | 0.72 | 0.51 | 14.29 | 79.25 | 0.15 | 1.77 | 0.35 | 0.99 | 0.03 | 1.87 | 99.93 | CS |
| 119 | DCT12:6-B | proto-porcelain | 0.68 | 0.60 | 15.46 | 77.39 | 0.16 | 2.02 | 0.42 | 1.02 | 0.04 | 2.19 | 99.98 | CS |
| 120 | DCT12:7-B | impressed stoneware | 0.53 | 0.46 | 15.58 | 77.67 | 0.11 | 1.54 | 0.30 | 1.24 | 0.02 | 2.48 | 99.93 | CS |
| 121 | DCT12:8-B | proto-porcelain | 0.58 | 0.39 | 13.69 | 80.33 | 0.22 | 1.55 | 0.32 | 1.00 | 0.02 | 1.86 | 99.96 | CS |
| 122 | DCT12:9-B | proto-porcelain | 0.72 | 0.68 | 17.78 | 74.53 | 0.17 | 1.87 | 0.45 | 1.00 | 0.03 | 2.76 | 99.99 | CS |
| 123 | DCT12:10-B | proto-porcelain | 0.64 | 0.54 | 16.13 | 77.15 | 0.14 | 1.84 | 0.36 | 1.01 | 0.03 | 2.15 | 99.99 | CS |
| 124 | DCT12:11-B | proto-porcelain | 0.75 | 0.58 | 14.45 | 79.25 | 0.16 | 1.57 | 0.39 | 0.98 | 0.01 | 1.81 | 99.95 | CS |
| 125 | DCT12:12-B | proto-porcelain | 0.79 | 0.48 | 14.44 | 79.22 | 0.21 | 1.59 | 0.39 | 0.93 | 0.03 | 1.85 | 99.93 | CS |
| 126 | DCT12:13-B | proto-porcelain | 0.65 | 0.47 | 13.26 | 80.56 | 0.21 | 1.70 | 0.36 | 0.98 | 0.02 | 1.78 | 99.99 | CS |
| 127 | DCT12:14-B | proto-porcelain | 0.76 | 0.62 | 15.48 | 77.01 | 0.14 | 1.93 | 0.47 | 1.03 | 0.03 | 2.51 | 99.98 | CS |
| 128 | DCT12:15-B | proto-porcelain | 0.61 | 0.64 | 15.97 | 77.00 | 0.05 | 1.85 | 0.39 | 1.00 | 0.03 | 2.45 | 99.99 | CS |
| 129 | DCT12:16-B | proto-porcelain | 0.50 | 0.52 | 14.60 | 79.24 | 0.10 | 1.82 | 0.28 | 1.02 | 0.02 | 1.87 | 99.97 | CS |
| 130 | DCT12:17-B | proto-porcelain | 0.60 | 0.60 | 16.81 | 75.89 | 0.10 | 1.95 | 0.40 | 1.15 | 0.03 | 2.45 | 99.98 | CS |
| 131 | DCT12:18-B | proto-porcelain | 0.33 | 0.50 | 16.95 | 76.25 | 0.08 | 1.97 | 0.41 | 1.01 | 0.02 | 2.42 | 99.94 | CS |
| 132 | DCT12:19-B | proto-porcelain | 0.62 | 0.56 | 15.75 | 77.29 | 0.17 | 1.94 | 0.39 | 1.03 | 0.02 | 2.16 | 99.93 | CS |
| 133 | DCT12:20-B | proto-porcelain | 0.67 | 0.74 | 16.36 | 75.94 | 0.14 | 1.94 | 0.32 | 1.11 | 0.03 | 2.65 | 99.90 | CS |
| 134 | DTT3035:1-B | proto-porcelain | 0.41 | 0.62 | 15.42 | 77.42 | 0.27 | 1.84 | 0.41 | 1.15 | 0.02 | 2.42 | 99.98 | TZQ |
| 135 | DTT3035:2-B | proto-porcelain | 0.45 | 0.57 | 15.24 | 77.53 | 0.15 | 1.90 | 0.40 | 1.22 | 0.03 | 2.49 | 99.98 | TZQ |
| 136 | DTT3035:3-B | proto-porcelain | 0.41 | 0.60 | 14.90 | 77.88 | 0.11 | 1.95 | 0.39 | 1.10 | 0.03 | 2.61 | 99.98 | TZQ |
| 137 | DTT3035:4-B | proto-porcelain | 0.39 | 0.58 | 14.83 | 78.09 | 0.11 | 1.90 | 0.41 | 1.16 | 0.02 | 2.50 | 99.99 | TZQ |
| 138 | DTT3035:5-B | proto-porcelain | 0.62 | 0.62 | 16.18 | 76.58 | 0.33 | 1.59 | 0.35 | 1.02 | 0.03 | 2.64 | 99.96 | TZQ |
| 139 | DTT3035:6-B | proto-porcelain | 0.39 | 0.72 | 16.15 | 76.52 | 0.15 | 1.67 | 0.28 | 1.10 | 0.02 | 2.90 | 99.90 | TZQ |
| 140 | DTT3035:7-B | proto-porcelain | 0.58 | 0.76 | 16.79 | 76.13 | 0.38 | 1.57 | 0.32 | 1.12 | 0.02 | 2.25 | 99.92 | TZQ |
| 141 | DTT3035:8-B | impressed stoneware | 0.47 | 0.66 | 16.48 | 76.32 | 0.34 | 1.75 | 0.33 | 1.18 | 0.02 | 2.44 | 99.99 | TZQ |
| 142 | DTT3035:9-B | proto-porcelain | 0.41 | 0.70 | 16.47 | 75.92 | 0.32 | 1.86 | 0.36 | 1.15 | 0.02 | 2.77 | 99.98 | TZQ |
| 143 | DTT3035:10-B | proto-porcelain | 0.66 | 0.52 | 16.94 | 75.72 | 0.17 | 1.74 | 0.43 | 1.03 | 0.02 | 2.74 | 99.97 | TZQ |
| 144 | DTT3035:11-B | proto-porcelain | 0.51 | 0.53 | 14.15 | 79.35 | 0.14 | 1.49 | 0.27 | 1.11 | 0.02 | 2.36 | 99.93 | TZQ |
| 145 | DTT3035:12-B | proto-porcelain | 0.43 | 0.53 | 14.39 | 78.71 | 0.14 | 1.69 | 0.31 | 1.15 | 0.02 | 2.54 | 99.91 | TZQ |
| 146 | DTT3035:13-B | proto-porcelain | 0.40 | 0.52 | 13.74 | 79.88 | 0.32 | 1.54 | 0.26 | 1.10 | 0.02 | 2.19 | 99.97 | TZQ |
| 147 | DTT3035:14-B | proto-porcelain | 0.30 | 0.61 | 15.54 | 77.00 | 0.20 | 1.99 | 0.39 | 1.12 | 0.02 | 2.77 | 99.94 | TZQ |
| 148 | DTT3035:15-B | proto-porcelain | 0.29 | 0.49 | 14.63 | 78.34 | 0.14 | 1.93 | 0.37 | 1.22 | 0.03 | 2.50 | 99.94 | TZQ |
| 149 | DTT3035:16-B | proto-porcelain | 0.30 | 0.60 | 15.65 | 77.16 | 0.21 | 1.93 | 0.36 | 1.13 | 0.01 | 2.58 | 99.93 | TZQ |
| 150 | DTT3035:17-B | proto-porcelain | 0.23 | 0.57 | 14.70 | 77.94 | 0.17 | 1.86 | 0.33 | 1.10 | 0.02 | 3.05 | 99.97 | TZQ |
| 151 | DTT3035:18-B | proto-porcelain | 0.43 | 0.63 | 16.02 | 76.30 | 0.08 | 2.09 | 0.43 | 1.24 | 0.03 | 2.61 | 99.86 | TZQ |
| 152 | DTT3035:19-B | proto-porcelain | 0.28 | 0.69 | 16.52 | 75.27 | 0.03 | 2.17 | 0.48 | 1.17 | 0.05 | 3.29 | 99.95 | TZQ |
| 153 | DTT3035:20-B | impressed stoneware | 0.51 | 0.73 | 17.01 | 76.23 | 0.03 | 1.73 | 0.33 | 1.16 | 0.00 | 2.20 | 99.93 | TZQ |
