# Supplementary material for: The Earliest Chinese Proto-Porcelain Excavated from Kiln Sites: An Elemental Analysis
Source: PLoS One. 2015 Nov 4;10(11):e0139970. doi: 10.1371/journal.pone.0139970 (PMC4633156; doi:10.1371/journal.pone.0139970)
Supplement: S2 Table — (DOC) [file pone.0139970.s006.doc]

**S2 Table. PIXE results of the chemical compositions (wt%) of the surfaces of impressed stoneware and** proto-porcelain sherds from 6 kiln sites.

| No. | Data number | Glazed | Na_2_O | MgO | Al_2_O_3_ | SiO_2_ | P_2_O_5_ | K_2_O | CaO | TiO_2_ | MnO | Fe_2_O_3_ | Total | Kiln |
| --- | --- | --- | --- | --- | --- | --- | --- | --- | --- | --- | --- | --- | --- | --- |
| 1 | HPT45:1-out | No | 0.62 | 1.47 | 15.91 | 67.44 | 0.20 | 5.00 | 2.68 | 1.03 | 0.09 | 5.45 | 99.89 | PS |
| 2 | HPT45:2-out | No | 0.41 | 1.33 | 17.66 | 68.37 | 0.33 | 3.78 | 1.54 | 1.09 | 0.07 | 5.29 | 99.87 | PS |
| 3 | HPT45:3-out | Yes | 0.49 | 1.29 | 16.38 | 67.05 | 0.32 | 3.63 | 4.92 | 1.06 | 0.10 | 4.57 | 99.81 | PS |
| 4 | HPT45:3-out | No | 0.42 | 1.31 | 16.96 | 67.33 | 0.38 | 3.27 | 2.46 | 1.30 | 0.07 | 6.34 | 99.84 | PS |
| 5 | HPT45:4-out | No | 0.83 | 1.23 | 15.04 | 70.18 | 0.31 | 4.48 | 2.06 | 1.08 | 0.07 | 4.55 | 99.83 | PS |
| 6 | HPT45:4-in | No | 0.38 | 0.69 | 18.85 | 72.24 | 0.12 | 2.37 | 0.26 | 1.17 | 0.04 | 3.77 | 99.89 | PS |
| 7 | HPT45:5-out | No | 0.58 | 0.97 | 16.16 | 72.04 | 0.13 | 4.13 | 1.17 | 1.00 | 0.02 | 3.76 | 99.96 | PS |
| 8 | HPT45:6-out | No | 0.36 | 1.04 | 16.55 | 62.90 | 0.40 | 3.78 | 2.48 | 1.07 | 0.47 | 10.77 | 99.82 | PS |
| 9 | HPT45:7-out | No | 0.54 | 1.31 | 15.90 | 69.24 | 0.32 | 3.77 | 2.21 | 0.93 | 0.08 | 5.65 | 99.95 | PS |
| 10 | HPT45:7-in | No | 0.21 | 0.55 | 20.47 | 71.47 | 0.04 | 1.26 | 0.22 | 1.04 | 0.05 | 4.63 | 99.94 | PS |
| 11 | HPT45:8-out | No | 0.50 | 1.57 | 17.90 | 65.46 | 0.29 | 3.57 | 3.36 | 1.08 | 0.14 | 6.13 | 100.00 | PS |
| 12 | HPT47:1-out | No | 0.36 | 1.29 | 16.06 | 68.20 | 0.45 | 3.24 | 2.86 | 1.03 | 0.14 | 6.26 | 99.89 | PS |
| 13 | HPT47:2-out | No | 0.32 | 1.04 | 20.04 | 68.29 | 0.17 | 3.47 | 0.72 | 1.09 | 0.04 | 4.78 | 99.96 | PS |
| 14 | HPT47:3-out | No | 0.18 | 0.95 | 16.39 | 72.46 | 0.19 | 2.61 | 1.45 | 1.33 | 0.05 | 4.30 | 99.91 | PS |
| 15 | HPT47:4-out | Yes | 0.53 | 1.51 | 14.19 | 65.92 | 0.41 | 3.11 | 7.08 | 0.98 | 0.17 | 5.99 | 99.89 | PS |
| 16 | HPT47:4-mouth site | No | 0.61 | 1.47 | 18.60 | 68.58 | 0.17 | 3.97 | 1.14 | 1.03 | 0.02 | 4.11 | 99.70 | PS |
| 17 | HPT47:5-out | No | 0.51 | 1.39 | 17.67 | 65.57 | 0.28 | 4.90 | 2.83 | 1.00 | 0.11 | 5.55 | 99.81 | PS |
| 18 | HPT47:6-out | No | 0.70 | 1.57 | 17.50 | 64.98 | 0.22 | 4.77 | 3.11 | 1.04 | 0.13 | 5.93 | 99.95 | PS |
| 19 | HPT47:7-out | No | 0.46 | 1.15 | 19.84 | 66.27 | 0.29 | 4.87 | 1.40 | 1.23 | 0.07 | 4.21 | 99.79 | PS |
| 20 | HPT47:8-out | No | 0.36 | 1.17 | 18.79 | 65.40 | 0.23 | 3.73 | 2.25 | 1.17 | 0.16 | 6.51 | 99.77 | PS |
| 21 | HPT47:9-out | No | 0.77 | 1.55 | 16.19 | 65.86 | 0.36 | 5.13 | 3.80 | 1.04 | 0.13 | 5.08 | 99.91 | PS |
| 22 | HBT13:1-in | No | 1.04 | 1.20 | 16.03 | 69.37 | 0.32 | 3.26 | 2.74 | 0.97 | 0.06 | 4.85 | 99.84 | BJS |
| 23 | HBT13:1-out | No | 0.87 | 1.06 | 17.13 | 72.59 | 0.31 | 2.77 | 0.66 | 0.89 | 0.05 | 3.63 | 99.96 | BJS |
| 24 | HBT13:2-in-G | Yes | 0.91 | 1.53 | 14.34 | 68.85 | 0.53 | 2.71 | 5.57 | 0.94 | 0.06 | 4.46 | 99.90 | BJS |
| 25 | HBT13:3-in-G | Yes | 0.86 | 1.30 | 14.25 | 69.27 | 0.53 | 2.81 | 5.27 | 1.00 | 0.26 | 4.43 | 99.98 | BJS |
| 26 | HBT13:4-in-G | No | 0.47 | 1.13 | 14.61 | 69.71 | 0.34 | 3.06 | 3.55 | 1.16 | 0.12 | 5.78 | 99.93 | BJS |
| 27 | HBT13:4-out | No | 0.72 | 1.11 | 16.50 | 68.72 | 0.20 | 6.00 | 0.85 | 1.04 | 0.05 | 4.67 | 99.86 | BJS |
| 28 | HBT13:5-G-in | Yes | 0.54 | 1.94 | 13.03 | 59.03 | 0.93 | 2.18 | 17.36 | 0.82 | 0.31 | 3.86 | 100.00 | BJS |
| 29 | HBT13:6-in-G | No | 0.82 | 1.18 | 16.00 | 70.17 | 0.50 | 2.87 | 3.53 | 0.94 | 0.09 | 3.90 | 100.00 | BJS |
| 30 | HBT13:7-in-G | Yes | 1.07 | 1.35 | 14.78 | 68.78 | 0.47 | 3.00 | 4.75 | 1.02 | 0.09 | 4.61 | 99.92 | BJS |
| 31 | HBT13:8-in-G | No | 0.52 | 1.08 | 15.38 | 72.83 | 0.33 | 2.63 | 0.98 | 1.13 | 0.09 | 5.01 | 99.98 | BJS |
| 32 | HBT13:9-in-G | Yes | 0.47 | 1.73 | 14.87 | 59.10 | 0.78 | 2.39 | 14.07 | 0.97 | 0.20 | 5.20 | 99.78 | BJS |
| 33 | HBT13:10-G-in | No | 0.41 | 1.52 | 14.57 | 70.22 | 0.43 | 2.50 | 2.98 | 1.19 | 0.12 | 6.02 | 99.96 | BJS |
| 34 | HBT13:15-G-out | Yes | 0.84 | 1.28 | 14.94 | 71.11 | 0.48 | 2.58 | 4.02 | 0.94 | 0.12 | 3.68 | 99.99 | BJS |
| 35 | HBT13:15-out-green site | Yes | 0.90 | 1.31 | 14.81 | 69.77 | 0.63 | 2.57 | 5.50 | 0.99 | 0.13 | 3.34 | 99.95 | BJS |
| 36 | HBT13:16-out | No | 1.21 | 0.98 | 15.62 | 70.38 | 0.51 | 3.78 | 2.27 | 1.01 | 0.08 | 4.12 | 99.96 | BJS |
| 37 | HBT13:17-out-G | Yes | 0.98 | 1.25 | 15.80 | 67.86 | 0.44 | 3.09 | 5.08 | 0.95 | 0.13 | 4.35 | 99.93 | BJS |
| 38 | HBT13:18-G-out | No | 1.53 | 1.53 | 14.16 | 67.72 | 0.35 | 5.45 | 4.59 | 0.79 | 0.11 | 3.61 | 99.84 | BJS |
| 39 | HBT13:19-G-out | No | 2.43 | 1.34 | 12.87 | 66.37 | 0.28 | 7.27 | 3.90 | 0.92 | 0.10 | 4.51 | 99.99 | BJS |
| 40 | HBT13:20-G-bottom | Yes | 0.88 | 1.23 | 15.80 | 65.41 | 0.62 | 2.75 | 5.86 | 0.78 | 0.10 | 6.53 | 99.96 | BJS |
| 41 | HNG1:1G-in | Yes | 0.93 | 1.19 | 15.59 | 61.96 | 0.32 | 3.15 | 11.85 | 1.12 | 0.13 | 3.49 | 99.73 | NS |
| 42 | HNG1:2G-in | No | 0.79 | 0.90 | 15.72 | 68.68 | 0.40 | 3.91 | 3.63 | 1.26 | 0.06 | 3.65 | 99.00 | NS |
| 43 | HNG1:3G-in | No | 1.41 | 1.48 | 17.08 | 64.79 | 0.66 | 5.04 | 4.64 | 1.00 | 0.12 | 3.33 | 99.55 | NS |
| 44 | HNG1:4G-in | Yes | 0.70 | 1.04 | 16.18 | 66.31 | 0.36 | 3.13 | 4.88 | 1.18 | 0.10 | 5.16 | 99.04 | NS |
| 45 | HNT4022:1-G | Yes | 1.03 | 1.50 | 15.63 | 65.41 | 0.52 | 2.50 | 8.96 | 1.03 | 0.18 | 2.72 | 99.48 | NS |
| 46 | HNT4022:2G | No | 2.35 | 1.13 | 18.95 | 66.18 | 0.08 | 5.08 | 1.44 | 0.86 | 0.07 | 3.41 | 99.55 | NS |
| 47 | HNT4028:1G-in | No | 1.74 | 0.97 | 15.44 | 69.68 | 0.60 | 3.82 | 2.78 | 0.97 | 0.13 | 3.77 | 99.90 | NS |
| 48 | HNT4028:2G | No | 2.52 | 0.88 | 18.25 | 68.84 | 0.32 | 3.30 | 2.46 | 0.78 | 0.11 | 2.39 | 99.85 | NS |
| 49 | HNT4042:1G | Yes | 0.87 | 0.77 | 11.48 | 62.85 | 1.58 | 2.33 | 15.28 | 1.00 | 0.24 | 3.16 | 99.56 | NS |
| 50 | HNT4042:2G-out | No | 1.88 | 0.97 | 15.84 | 69.51 | 0.22 | 5.13 | 2.01 | 1.12 | 0.13 | 2.90 | 99.71 | NS |
| 51 | HNT4043:1G | Yes | 1.89 | 1.59 | 15.77 | 59.19 | 0.59 | 3.53 | 12.64 | 0.94 | 0.18 | 3.52 | 99.84 | NS |
| 52 | HNT4044:1G-in | Yes | 1.39 | 1.01 | 15.26 | 67.59 | 0.66 | 4.12 | 4.43 | 1.17 | 0.11 | 4.23 | 99.97 | NS |
| 53 | HNT4045:1G-out | Yes | 1.44 | 2.13 | 14.29 | 57.02 | 0.80 | 3.41 | 16.60 | 0.91 | 0.19 | 3.03 | 99.82 | NS |
| 54 | HNT4045:2G-out | No | 1.45 | 0.90 | 14.48 | 69.65 | 0.39 | 3.63 | 3.82 | 0.95 | 0.23 | 4.37 | 99.87 | NS |
| 55 | HNT4045:2G-in | No | 2.48 | 0.81 | 15.57 | 65.77 | 0.29 | 6.47 | 1.83 | 0.95 | 0.13 | 5.67 | 99.97 | NS |
| 56 | HNT4046:1G-out | No | 2.14 | 0.78 | 16.06 | 66.62 | 0.09 | 6.64 | 1.32 | 1.03 | 0.13 | 4.89 | 99.70 | NS |
| 57 | HNT4046:1G-in | No | 1.24 | 0.88 | 11.43 | 72.61 | 0.37 | 5.19 | 1.32 | 0.86 | 0.16 | 3.76 | 97.82 | NS |
| 58 | HNT4046:2-G-out | No | 3.26 | 0.71 | 13.81 | 68.82 | 0.00 | 6.64 | 1.46 | 1.03 | 0.10 | 3.89 | 99.72 | NS |
| 59 | HNT4046:2-G-in | Yes | 1.89 | 1.41 | 13.13 | 59.95 | 0.82 | 3.94 | 12.74 | 0.99 | 0.36 | 4.33 | 99.56 | NS |
| 60 | HNT4047:1-G-in | No | 1.31 | 0.97 | 17.48 | 70.35 | 0.33 | 3.28 | 1.89 | 0.95 | 0.07 | 3.16 | 99.79 | NS |
| 61 | HNT4047:2-G-in | Yes | 0.91 | 1.01 | 17.60 | 65.25 | 0.43 | 3.34 | 5.14 | 1.11 | 0.12 | 4.81 | 99.72 | NS |
| 62 | HNT4047:2-G-out | No | 0.44 | 1.07 | 24.01 | 60.96 | 0.36 | 3.52 | 0.74 | 1.23 | 0.08 | 7.16 | 99.57 | NS |
| 63 | HNT4047:3-G-in | Yes | 0.83 | 1.13 | 16.92 | 64.77 | 0.70 | 2.65 | 7.71 | 1.26 | 0.15 | 3.65 | 99.77 | NS |
| 64 | HNT4047:3-G-out | No | 0.71 | 0.88 | 23.43 | 61.97 | 0.15 | 3.87 | 0.87 | 1.03 | 0.07 | 6.78 | 99.76 | NS |
| 65 | HNT4047:4-G-out | Yes | 0.91 | 1.81 | 14.27 | 57.81 | 0.84 | 2.11 | 17.81 | 0.97 | 0.48 | 2.75 | 99.76 | NS |
| 66 | DHT5045:1-G-out | Yes | 0.36 | 2.22 | 16.16 | 58.95 | 1.10 | 1.44 | 13.63 | 0.75 | 0.07 | 5.23 | 99.91 | HSS1 |
| 67 | DHT5045:2-G-out | Yes | 0.47 | 1.69 | 15.66 | 59.51 | 1.02 | 2.63 | 14.36 | 0.61 | 0.04 | 3.84 | 99.83 | HSS1 |
| 68 | DHT5045:3-G-in | Yes | 0.62 | 2.25 | 14.56 | 57.40 | 1.39 | 2.65 | 15.76 | 0.75 | 0.25 | 4.21 | 99.84 | HSS1 |
| 69 | DHT5045:4-G-out | Yes | 0.40 | 1.80 | 15.89 | 58.75 | 1.26 | 2.60 | 14.74 | 0.59 | 0.06 | 3.74 | 99.83 | HSS1 |
| 70 | DHT5045:4-G-in | Yes | 0.32 | 1.63 | 14.25 | 57.73 | 0.56 | 3.97 | 15.37 | 0.80 | 0.19 | 5.09 | 99.91 | HSS1 |
| 71 | DHT5045:5-G-out | Yes | 0.61 | 1.48 | 12.64 | 59.80 | 1.81 | 3.18 | 16.88 | 0.89 | 0.07 | 2.51 | 99.87 | HSS1 |
| 72 | DHT5045:6-G-out | Yes | 0.56 | 1.46 | 17.10 | 58.35 | 0.59 | 2.49 | 15.09 | 0.70 | 0.06 | 3.50 | 99.90 | HSS1 |
| 73 | DHT5045:6-G-in | Yes | 0.57 | 1.71 | 16.05 | 59.05 | 0.34 | 3.84 | 13.69 | 0.81 | 0.14 | 3.67 | 99.87 | HSS1 |
| 74 | DHT5045:7-G-out | Yes | 1.07 | 2.19 | 14.00 | 61.38 | 1.28 | 2.54 | 13.46 | 0.72 | 0.05 | 3.03 | 99.72 | HSS1 |
| 75 | DHT5045:8-G-out | Yes | 0.39 | 2.00 | 12.02 | 55.71 | 1.18 | 2.97 | 20.22 | 0.74 | 0.19 | 4.44 | 99.86 | HSS1 |
| 76 | DHT5045:9-G-out | Yes | 0.53 | 1.65 | 13.51 | 58.79 | 1.00 | 2.47 | 15.52 | 0.96 | 0.12 | 5.37 | 99.92 | HSS1 |
| 77 | DHT5045:10-G-out | Yes | 0.42 | 1.55 | 15.54 | 55.80 | 0.64 | 3.78 | 17.19 | 0.78 | 0.06 | 4.14 | 99.90 | HSS1 |
| 78 | DHT5045:10-G-in | Yes | 0.26 | 1.66 | 12.97 | 56.83 | 0.63 | 3.15 | 18.66 | 0.89 | 0.31 | 4.57 | 99.93 | HSS1 |
| 79 | DHT5045:11-G-out | Yes | 0.73 | 2.68 | 17.39 | 58.37 | 0.80 | 3.60 | 10.90 | 0.83 | 0.12 | 4.52 | 99.94 | HSS1 |
| 80 | DHT5045:12-G-out | Yes | 0.57 | 2.19 | 17.87 | 60.37 | 0.69 | 2.90 | 8.80 | 0.90 | 0.11 | 5.51 | 99.91 | HSS1 |
| 81 | DHT5045:13-G-out | Yes | 0.47 | 1.93 | 17.21 | 59.17 | 0.60 | 2.71 | 13.63 | 0.76 | 0.11 | 3.36 | 99.95 | HSS1 |
| 82 | DHT5045:14-G-out | Yes | 0.74 | 1.85 | 16.18 | 58.62 | 0.72 | 2.59 | 14.89 | 0.75 | 0.07 | 3.47 | 99.88 | HSS1 |
| 83 | DHT5045:15-G-out | Yes | 0.82 | 2.00 | 15.92 | 57.13 | 0.87 | 3.25 | 15.55 | 0.61 | 0.03 | 3.73 | 99.91 | HSS1 |
| 84 | DHT5045:16-G-out | Yes | 0.82 | 2.72 | 13.92 | 57.80 | 1.05 | 2.27 | 16.83 | 0.69 | 0.11 | 3.49 | 99.70 | HSS1 |
| 85 | DHT5045:17-G-out | Yes | 0.60 | 2.47 | 14.66 | 61.32 | 1.28 | 1.68 | 13.08 | 0.75 | 0.14 | 3.77 | 99.75 | HSS1 |
| 86 | DHT5045:18-G-out | Yes | 0.46 | 1.57 | 11.20 | 57.82 | 1.37 | 3.23 | 17.57 | 0.91 | 0.10 | 5.66 | 99.89 | HSS1 |
| 87 | DHT5045:19-G-out | Yes | 0.47 | 1.85 | 13.95 | 62.17 | 0.92 | 2.23 | 12.49 | 1.01 | 0.15 | 4.73 | 99.97 | HSS1 |
| 88 | DHT5045:20-G-out | Yes | 0.75 | 2.30 | 16.33 | 59.25 | 0.97 | 2.95 | 12.12 | 0.83 | 0.11 | 4.35 | 99.96 | HSS1 |
| 89 | DHIIT3036:1-G-in | Yes | 0.70 | 1.74 | 12.67 | 69.34 | 0.64 | 3.14 | 7.96 | 0.86 | 0.35 | 2.50 | 99.90 | HSS2 |
| 90 | DHIIT3036:2-G-in | Yes | 0.75 | 2.49 | 11.69 | 60.07 | 1.13 | 2.21 | 18.04 | 0.88 | 0.37 | 2.30 | 99.93 | HSS2 |
| 91 | DHIIT3036:3-G | Yes | 0.59 | 2.19 | 12.84 | 59.40 | 1.08 | 2.44 | 17.49 | 0.84 | 0.14 | 2.90 | 99.91 | HSS2 |
| 92 | DHIIT3036:4-G-in | Yes | 0.88 | 2.35 | 13.80 | 65.30 | 0.96 | 2.71 | 10.58 | 0.78 | 0.21 | 2.43 | 100.00 | HSS2 |
| 93 | DHIIT3036:5-G-in | Yes | 0.60 | 2.04 | 14.92 | 64.91 | 0.64 | 2.08 | 10.93 | 0.84 | 0.23 | 2.77 | 99.96 | HSS2 |
| 94 | DHIIT3036:6-G-out | Yes | 0.63 | 2.25 | 13.14 | 58.20 | 2.03 | 2.67 | 18.00 | 0.78 | 0.07 | 2.13 | 99.90 | HSS2 |
| 95 | DHIIT3036:7-G-out | Yes | 0.63 | 2.47 | 15.56 | 63.27 | 1.55 | 2.26 | 10.84 | 0.69 | 0.39 | 2.22 | 99.88 | HSS2 |
| 96 | DHIIT3036:8-G | Yes | 1.06 | 1.87 | 13.75 | 65.59 | 0.96 | 3.24 | 10.11 | 0.81 | 0.26 | 2.30 | 99.95 | HSS2 |
| 97 | DHIIT3036:9-G-out | Yes | 1.26 | 1.25 | 15.66 | 68.06 | 0.89 | 4.25 | 4.98 | 0.79 | 0.21 | 2.58 | 99.93 | HSS2 |
| 98 | DHIIT3036:10-G-in | Yes | 0.70 | 2.02 | 12.61 | 63.03 | 1.06 | 2.17 | 13.94 | 1.09 | 0.25 | 2.99 | 99.86 | HSS2 |
| 99 | DHIIT3036:11-G-mouth site | Yes | 1.06 | 1.53 | 15.94 | 67.60 | 0.60 | 4.78 | 4.97 | 0.79 | 0.26 | 2.47 | 100.00 | HSS2 |
| 100 | DHIIT3036:12-G-in | Yes | 0.46 | 1.46 | 13.07 | 68.05 | 0.71 | 2.51 | 10.18 | 0.85 | 0.22 | 2.44 | 99.95 | HSS2 |
| 101 | DHIIT3036:13-G-in-bottom | Yes | 0.68 | 1.27 | 13.32 | 68.63 | 0.85 | 2.86 | 8.54 | 1.01 | 0.24 | 2.50 | 99.90 | HSS2 |
| 102 | DHIIT3036:14-G-out | Yes | 0.78 | 1.93 | 14.18 | 60.55 | 0.98 | 2.44 | 15.17 | 0.88 | 0.15 | 2.78 | 99.84 | HSS2 |
| 103 | DHIIT3036:15-G-in | Yes | 0.81 | 2.37 | 12.39 | 57.39 | 1.47 | 2.59 | 19.58 | 0.80 | 0.30 | 2.18 | 99.88 | HSS2 |
| 104 | DHIIT3036:16-G-out | Yes | 0.68 | 1.87 | 14.58 | 59.51 | 1.66 | 2.03 | 16.23 | 0.78 | 0.18 | 2.41 | 99.93 | HSS2 |
| 105 | DHIIT3036:17-G | Yes | 0.79 | 3.04 | 13.86 | 56.60 | 1.59 | 2.59 | 17.78 | 0.83 | 0.33 | 2.54 | 99.95 | HSS2 |
| 106 | DHIIT3036:18-G-out | Yes | 1.00 | 2.89 | 12.78 | 57.02 | 1.68 | 2.17 | 19.14 | 0.72 | 0.60 | 1.87 | 99.87 | HSS2 |
| 107 | DHIIT3036:19-G-in | Yes | 0.48 | 1.26 | 12.33 | 68.20 | 0.79 | 1.63 | 10.89 | 0.85 | 0.24 | 3.18 | 99.85 | HSS2 |
| 108 | DHIIT3036:20-G-in | Yes | 0.73 | 1.35 | 16.03 | 68.10 | 0.81 | 2.88 | 6.58 | 0.67 | 0.24 | 2.55 | 99.94 | HSS2 |
| 109 | DHIIIT3032:1-G-in | Yes | 0.74 | 1.71 | 12.85 | 63.40 | 0.73 | 2.35 | 13.85 | 0.85 | 0.88 | 2.60 | 99.96 | HSS3 |
| 110 | DHIIIT3032:2-G-in | Yes | 0.92 | 1.66 | 13.33 | 64.81 | 0.67 | 2.46 | 12.76 | 0.95 | 0.23 | 2.05 | 99.84 | HSS3 |
| 111 | DHIIIT3032:3-G-out | Yes | 1.35 | 1.35 | 15.30 | 68.96 | 0.66 | 4.18 | 4.84 | 0.91 | 0.13 | 2.22 | 99.90 | HSS3 |
| 112 | DHIIIT3032:4-G-out | Yes | 0.80 | 1.95 | 14.57 | 61.44 | 1.34 | 2.20 | 14.35 | 0.82 | 0.19 | 2.27 | 99.93 | HSS3 |
| 113 | DHIIIT3032:5-G-out | Yes | 1.11 | 1.50 | 14.52 | 62.95 | 1.10 | 3.32 | 11.72 | 0.95 | 0.25 | 2.52 | 99.94 | HSS3 |
| 114 | DHIIIT3032:6-G-in | Yes | 0.85 | 1.67 | 12.69 | 63.97 | 0.81 | 2.36 | 14.06 | 0.90 | 0.22 | 2.37 | 99.90 | HSS3 |
| 115 | DHIIIT3032:7-G-out | Yes | 0.80 | 1.23 | 15.20 | 67.22 | 0.40 | 3.20 | 7.97 | 0.92 | 0.17 | 2.69 | 99.80 | HSS3 |
| 116 | DHIIIT3032:8-G-in | Yes | 0.66 | 1.85 | 12.91 | 60.93 | 1.40 | 2.19 | 15.74 | 1.04 | 0.21 | 3.04 | 99.97 | HSS3 |
| 117 | DHIIIT3032:9-G | Yes | 0.86 | 1.73 | 15.37 | 63.52 | 0.58 | 2.22 | 12.04 | 0.87 | 0.14 | 2.62 | 99.95 | HSS3 |
| 118 | DHIIIT3032:10-G-out | Yes | 0.85 | 2.33 | 13.77 | 61.15 | 1.07 | 2.03 | 15.25 | 0.88 | 0.27 | 2.39 | 99.99 | HSS3 |
| 119 | DHIIIT3032:11-G-out | Yes | 0.95 | 2.62 | 13.92 | 58.50 | 1.22 | 2.29 | 17.24 | 0.87 | 0.17 | 2.15 | 99.93 | HSS3 |
| 120 | DHIIIT3032:12-G-out | Yes | 1.09 | 2.07 | 15.76 | 63.85 | 0.98 | 2.50 | 10.36 | 0.90 | 0.09 | 2.35 | 99.95 | HSS3 |
| 121 | DHIIIT3032:13-G-out | Yes | 0.80 | 1.56 | 15.82 | 63.13 | 0.58 | 2.33 | 11.69 | 0.91 | 0.14 | 2.98 | 99.94 | HSS3 |
| 122 | DHIIIT3032:14-G-in | Yes | 0.96 | 2.02 | 11.23 | 58.70 | 1.33 | 2.35 | 19.72 | 0.80 | 0.48 | 2.25 | 99.84 | HSS3 |
| 123 | DHIIIT3032:15-G-in | Yes | 0.85 | 1.85 | 13.39 | 63.75 | 1.00 | 2.50 | 12.35 | 1.02 | 0.25 | 2.96 | 99.92 | HSS3 |
| 124 | DHIIIT3032:16-G-in | Yes | 0.90 | 2.56 | 10.95 | 56.65 | 1.56 | 2.10 | 21.24 | 0.82 | 0.58 | 2.55 | 99.91 | HSS3 |
| 125 | DHIIIT3032:17-G-out | Yes | 0.82 | 1.55 | 15.13 | 63.60 | 0.71 | 2.96 | 11.40 | 1.00 | 0.25 | 2.46 | 99.88 | HSS3 |
| 126 | DHIIIT3032:18-G-out | Yes | 0.89 | 1.55 | 13.16 | 67.40 | 0.86 | 2.95 | 9.03 | 0.96 | 0.40 | 2.70 | 99.90 | HSS3 |
| 127 | DHIIIT3032:19-G | Yes | 0.92 | 2.19 | 13.47 | 61.85 | 1.40 | 2.65 | 13.25 | 0.89 | 0.21 | 3.08 | 99.91 | HSS3 |
| 128 | DHIIIT3032:20-G-out | Yes | 2.21 | 1.93 | 14.85 | 61.14 | 0.70 | 6.05 | 9.44 | 0.85 | 0.20 | 2.59 | 99.96 | HSS3 |
| 129 | DCT12:1-G-out | Yes | 0.60 | 3.34 | 10.96 | 57.81 | 1.91 | 1.68 | 19.40 | 0.81 | 0.61 | 2.47 | 99.59 | CS |
| 130 | DCT12:2-G-out | Yes | 0.70 | 2.09 | 11.61 | 63.30 | 1.15 | 1.67 | 15.64 | 0.90 | 0.58 | 2.32 | 99.96 | CS |
| 131 | DCT12:3-G-out | Yes | 0.71 | 2.87 | 11.68 | 58.57 | 2.06 | 1.39 | 18.29 | 0.84 | 0.37 | 3.07 | 99.85 | CS |
| 132 | DCT12:4-G-in | Yes | 0.45 | 2.33 | 10.79 | 61.40 | 1.18 | 0.99 | 18.86 | 0.91 | 0.45 | 2.62 | 99.98 | CS |
| 133 | DCT12:5-G | Yes | 0.64 | 2.63 | 11.16 | 60.58 | 1.34 | 0.98 | 19.75 | 0.77 | 0.36 | 1.64 | 99.85 | CS |
| 134 | DCT12:6-G | Yes | 0.63 | 2.38 | 13.33 | 60.44 | 1.14 | 1.72 | 16.99 | 0.88 | 0.05 | 2.39 | 99.95 | CS |
| 135 | DCT12:7-G-out | No | 1.02 | 1.71 | 14.36 | 68.29 | 0.84 | 3.54 | 6.21 | 1.09 | 0.05 | 2.87 | 99.98 | CS |
| 136 | DCT12:8-G | Yes | 0.62 | 2.16 | 11.47 | 62.82 | 1.29 | 1.49 | 15.79 | 0.88 | 0.28 | 3.13 | 99.93 | CS |
| 137 | DCT12:9-G-out | Yes | 0.78 | 3.27 | 12.51 | 60.49 | 1.57 | 1.47 | 15.19 | 0.86 | 0.55 | 3.21 | 99.90 | CS |
| 138 | DCT12:10-G-out | Yes | 1.01 | 2.30 | 12.41 | 59.40 | 0.99 | 2.91 | 17.02 | 0.90 | 0.43 | 2.47 | 99.84 | CS |
| 139 | DCT12:11-G-in | Yes | 0.77 | 2.22 | 12.08 | 60.99 | 1.07 | 2.12 | 17.51 | 0.84 | 0.34 | 1.90 | 99.84 | CS |
| 140 | DCT12:12-G | Yes | 1.05 | 2.34 | 11.41 | 59.79 | 1.18 | 2.38 | 17.59 | 0.89 | 0.40 | 2.13 | 99.16 | CS |
| 141 | DCT12:13-G-out | Yes | 0.80 | 2.09 | 10.66 | 60.53 | 1.28 | 2.25 | 18.24 | 0.84 | 0.42 | 2.85 | 99.96 | CS |
| 142 | DCT12:14-G | Yes | 0.77 | 3.59 | 10.16 | 54.49 | 2.01 | 1.78 | 22.55 | 0.75 | 0.77 | 3.04 | 99.91 | CS |
| 143 | DCT12:15-G | Yes | 0.80 | 2.30 | 13.03 | 62.83 | 1.09 | 3.12 | 12.51 | 0.95 | 0.32 | 2.93 | 99.88 | CS |
| 144 | DCT12:16-G-in | Yes | 0.49 | 2.20 | 10.48 | 60.70 | 1.04 | 1.61 | 20.24 | 0.81 | 0.27 | 2.09 | 99.93 | CS |
| 145 | DCT12:17-G-out | Yes | 0.74 | 3.27 | 11.92 | 58.06 | 1.77 | 1.79 | 18.28 | 0.87 | 0.46 | 2.76 | 99.92 | CS |
| 146 | DCT12:18-G-out | Yes | 0.62 | 2.86 | 12.76 | 59.24 | 1.51 | 1.54 | 17.36 | 0.85 | 0.41 | 2.75 | 99.90 | CS |
| 147 | DCT12:19-G-out | Yes | 0.50 | 2.57 | 12.50 | 59.76 | 1.23 | 1.57 | 17.88 | 0.92 | 0.42 | 2.65 | 100.00 | CS |
| 148 | DCT12:20-G-out | Yes | 0.34 | 2.52 | 12.71 | 60.49 | 1.03 | 1.55 | 17.29 | 0.99 | 0.45 | 2.54 | 99.91 | CS |
| 149 | DTT3035:1-G-out | Yes | 0.43 | 2.23 | 12.23 | 61.52 | 1.16 | 1.94 | 16.32 | 0.98 | 0.44 | 2.65 | 99.90 | TZQ |
| 150 | DTT3035:2-G-out | Yes | 0.40 | 2.09 | 11.42 | 59.54 | 1.34 | 1.68 | 18.93 | 1.03 | 0.53 | 2.99 | 99.95 | TZQ |
| 151 | DTT3035:3-G-out | Yes | 0.31 | 2.01 | 12.52 | 59.47 | 1.34 | 1.71 | 17.86 | 1.10 | 0.52 | 3.11 | 99.95 | TZQ |
| 152 | DTT3035:4-G-out | Yes | 0.36 | 2.11 | 11.43 | 57.04 | 1.33 | 1.86 | 19.55 | 1.07 | 0.73 | 4.42 | 99.90 | TZQ |
| 153 | DTT3035:5-G-out | Yes | 0.41 | 2.60 | 13.37 | 59.26 | 1.24 | 2.11 | 16.96 | 0.82 | 0.42 | 2.69 | 99.88 | TZQ |
| 154 | DTT3035:6-G-out | Yes | 0.58 | 2.22 | 12.64 | 60.94 | 0.94 | 2.67 | 13.95 | 1.13 | 0.81 | 4.02 | 99.90 | TZQ |
| 155 | DTT3035:7-G-out | Yes | 0.81 | 3.02 | 13.72 | 59.31 | 1.17 | 1.97 | 16.54 | 0.79 | 0.41 | 2.05 | 99.79 | TZQ |
| 156 | DTT3035:8-out | No | 0.93 | 1.74 | 14.88 | 70.42 | 0.61 | 2.66 | 4.85 | 0.77 | 0.18 | 2.62 | 99.66 | TZQ |
| 157 | DTT3035:9-G-out | Yes | 0.53 | 2.04 | 13.45 | 60.85 | 0.65 | 2.69 | 13.67 | 1.08 | 0.35 | 4.47 | 99.78 | TZQ |
| 158 | DTT3035:10-G-in | Yes | 0.37 | 2.20 | 11.05 | 53.66 | 1.24 | 1.55 | 23.71 | 1.01 | 0.80 | 4.21 | 99.80 | TZQ |
| 159 | DTT3035:11-G-in | Yes | 0.72 | 2.45 | 11.68 | 58.88 | 1.25 | 2.15 | 18.53 | 0.94 | 0.35 | 2.81 | 99.76 | TZQ |
| 160 | DTT3035:12-G-in | Yes | 0.38 | 1.83 | 9.24 | 59.84 | 0.88 | 1.63 | 18.36 | 1.05 | 0.86 | 5.80 | 99.87 | TZQ |
| 161 | DTT3035:13-G-in | Yes | 0.36 | 1.93 | 9.76 | 59.52 | 1.35 | 2.11 | 19.44 | 1.02 | 0.46 | 3.91 | 99.86 | TZQ |
| 162 | DTT3035:14-G-out | Yes | 0.61 | 2.18 | 13.02 | 61.90 | 0.95 | 1.58 | 14.78 | 1.01 | 0.58 | 3.26 | 99.87 | TZQ |
| 163 | DTT3035:15-G-out | Yes | 0.57 | 2.05 | 12.27 | 63.77 | 0.97 | 2.31 | 14.25 | 1.02 | 0.27 | 2.50 | 99.98 | TZQ |
| 164 | DTT3035:16-G-out | Yes | 0.45 | 1.79 | 11.64 | 63.39 | 0.72 | 2.14 | 14.41 | 1.01 | 0.41 | 3.85 | 99.81 | TZQ |
| 165 | DTT3035:17-G-out | Yes | 0.42 | 2.14 | 12.40 | 61.46 | 1.29 | 2.25 | 13.56 | 1.04 | 0.48 | 4.90 | 99.94 | TZQ |
| 166 | DTT3035:18-G-out | Yes | 0.81 | 1.75 | 12.60 | 61.74 | 0.64 | 3.76 | 15.35 | 0.96 | 0.21 | 2.09 | 99.91 | TZQ |
| 167 | DTT3035:19-G-in | Yes | 0.36 | 2.66 | 11.80 | 55.99 | 1.04 | 1.40 | 21.45 | 1.00 | 0.84 | 3.38 | 99.92 | TZQ |
| 168 | DTT3035:20-G-in | No | 0.33 | 0.72 | 9.91 | 81.96 | 0.30 | 1.80 | 1.49 | 1.14 | 0.05 | 2.14 | 99.84 | TZQ |
